# Viral Membrane Fusion and Nucleocapsid Delivery into the Cytoplasm are Distinct Events in Some Flaviviruses

**DOI:** 10.1371/journal.ppat.1003585

**Published:** 2013-09-05

**Authors:** Adel M. Nour, Yue Li, Joseph Wolenski, Yorgo Modis

**Affiliations:** 1 Department of Molecular Biophysics and Biochemistry, Yale University, New Haven, Connecticut, United States of America; 2 Department of Molecular, Cellular and Developmental Biology, Yale University, New Haven, Connecticut, United States of America; NIH, United States of America

## Abstract

Flaviviruses deliver their genome into the cell by fusing the viral lipid membrane to an endosomal membrane. The sequence and kinetics of the steps required for nucleocapsid delivery into the cytoplasm remain unclear. Here we dissect the cell entry pathway of virions and virus-like particles from two flaviviruses using single-particle tracking in live cells, a biochemical membrane fusion assay and virus infectivity assays. We show that the virus particles fuse with a small endosomal compartment in which the nucleocapsid remains trapped for several minutes. Endosomal maturation inhibitors inhibit infectivity but not membrane fusion. We propose a flavivirus cell entry mechanism in which the virus particles fuse preferentially with small endosomal carrier vesicles and depend on back-fusion of the vesicles with the late endosomal membrane to deliver the nucleocapsid into the cytoplasm. Virus entry modulates intracellular calcium release and phosphatidylinositol-3-phosphate kinase signaling. Moreover, the broadly cross-reactive therapeutic antibody scFv11 binds to virus-like particles and inhibits fusion.

## Author Summary

Many viruses package their genetic material into a lipid envelope. In order to deliver their genome into the host-cell cytoplasm, where it can be replicated, viruses must fuse their envelope with a cellular lipid membrane. This fusion event is therefore a critical step in the entry of an enveloped virus into the cell. In this study, we used various cell biological and biochemical approaches to map precisely the cell entry pathway of two major human pathogens from the flavivirus family, yellow fever virus and Japanese encephalitis virus. We discovered that these viruses co-opt cellular phospholipid signaling to promote the fusion of their envelope with the lipid envelope of small compartments inside the host-cell endosomes. The viral genome remains trapped in these compartments for several minutes until the compartments fuse with the surrounding endosomal membrane. It is this second membrane fusion event that delivers the viral genome into the cytoplasm. We also showed that the antibody fragment scFv11 inhibits the fusion of the viral envelope with small lipid compartments, explaining the therapeutic activity of the scFv11 antibody. Our work identifies new vulnerabilities in the entry pathway of flaviviruses, including the formation of small endosomal compartments and two distinct membrane fusion events involving these compartments.

## Introduction

Many enveloped RNA viruses utilize the endocytic pathway to enter host cells [1], [2]. Endocytosis begins at the cell membrane, where these viruses bind to their cellular receptors and ends at the lysosome, the “stomach” of the cell. Along the endocytic pathway, changes in the lipid composition and environmental pH provide a series of distinct milieus for specific cellular or viral functions to occur [3]. Enveloped viruses and bacterial toxins enter the endocytic pathway by binding receptors on the cell surface that are coupled to the endocytic machinery, in particular clathrin adaptors. These microbial cargoes undergo sorting at two different checkpoints [4], [5], [6]. The first is in early endosomes (EEs) where the vesicular contents are either directed back to the cell membrane via tubular structures, or targeted to the trans-Golgi network (TGN). Alternatively, the cargo contents are sorted into intraluminal vesicles and transported to late endosomes via endosomal carrier vesicles (ECVs). ECVs require functional microtubules to be transported to the second sorting station, the late endosomes. In late endosomes, cargo contents can be forwarded to the TGN, the cytoplasm, or for lysosomal degradation. ECVs originate from EEs. Both the EEs and ECVs are rich in cholesterol, phosphatidylserine (PS) and phosphatidylinositols (PI) [7], [8], [9]. The level of cholesterol decreases along the endocytic pathway and is replaced with ceramide in late endosomes and lysosomes, where it maintains membrane fluidity [10]. Unlike cholesterol and PS, the anionic lipid BMP (bis(monoacylglycero)phosphate), also known as LBPA (lysobisphosphatidic acid), is abundant in internal membranes of lysosomes and late endosomes, and depleted in the EEs [7]. BMP regulates membrane sorting and dynamics in the late endosome. Autoantibodies against this lipid result in human disorders such as Niemann-Pick type C (NPC) syndrome, characterized by dysfunctional sorting and trafficking in late endosomes [11].

The genus flavivirus includes important human pathogens such as dengue, Japanese encephalitis (JE), West Nile (WN) and yellow fever (YF) viruses. Flaviviruses contain a lipid envelope and a positive-stranded RNA genome encoding for a polyprotein that is processed by the host- and viral proteases to yield the viral proteins. Three structural proteins (C, M and E) form the virions; the nonstructural proteins (NS1-5) are required for virus replication, transcription and modulation of the host innate immune system [12]. Flaviviruses assemble in specialized structures within the endoplasmic reticulum and mature in the Golgi network [13]. Glycoprotein E forms the outer protein shell of the virion, mediates cellular attachment, and catalyzes the fusion of the viral and cellular membranes necessary to deliver the genome into the cytoplasm. The E ectodomain contains three domains (I–III) connected by hinges [14], [15]. Conserved histidine residues at the domain I-domain III interface become protonated at the reduced pH of early endosomal compartments (pH 6–6.5), thereby triggering a conformational rearrangement in E that drives membrane fusion [16], [17].

Although flaviviruses generally follow the clathrin-mediated endocytic pathway, other mechanisms of entry have also been proposed. Dengue virus has been reported to fuse primarily from within Rab7-positive late endosomes. However, certain dengue strains have been reported to infect cell independently of Rab7 [18] and alternative entry pathways have been proposed for specific viruses and cell types [19], [20]. Moreover, in certain flaviviruses low pH does not appear to be sufficient to trigger fusion suggesting that additional factors may be required [21]. Indeed, different compartment-specific lipids are required for fusion of dengue and Japanese encephalitis viruses [22], [23], [24], [25].

Flaviviruses produce small noninfectious virus-like particles (VLPs) during infection [26]. Little is known about the role of these VLPs in infection or in host immunity however, recombinant flavivirus VLPs are the subject of intense study as vaccine candidates and gene delivery vehicles [27], [28], [29], [30]. It is not known whether VLPs have the same requirements for fusion and the same cell entry pathways as full-sized virions.

In this study, we use JE-VLPs and YFV virions as model systems to study the cell flavivirus entry pathway. Using a combination of approaches—including single-particle tracking in live cells, a liposome-based membrane fusion assay, a quantitative RT-PCR RNA delivery assay, and viral infectivity assays—we show that our model viruses modulate cellular signal transduction to promote membrane fusion to ECVs, which occurs several minutes before nucleocapsid delivery into the cytoplasm, suggesting that these are two distinct events in virus entry. Our observations are consistent with an entry pathway in which certain flaviviruses fuse with ECVs and require host proteins to deliver the nucleocapsid to the cytoplasm. This pathway has a precedent in vesicular stomatitis virus (VSV) [5], although VSV does not impact PI-3-phosphate kinase activity [31], [32] nor trigger intracellular calcium release during entry [33]. Moreover, we demonstrated the ability of a broadly cross-reactive therapeutic antibody, scFv11, to block membrane fusion with the host cells and EE/ECV-like liposomes.

## Results

### Purification of Japanese encephalitis virus-like particles and yellow fever virus

Recombinant Japanese encephalitis virus-like particles (JE-VLPs) were obtained by overexpressing the prM and E genes (see Materials and Methods) in human HEK 293T cells (Figure S1A), and in insect Tni cells using a baculovirus-based expression system (Figure S1B). JE-VLPs and YFV were purified by precipitation of secreted cellular products with polyethylene glycol, followed by sedimentation in sucrose density gradient (Figure S2). Since flaviviruses E proteins bind to heparan sulfate [34], the virus particles could alternatively be purified by affinity chromatography on a heparan sulfate column (Figure S2A–B). Both methods showed highly purified secreted VLPs. Coomassie stained SDS-PAGE confirmed that prM was cleaved to pr and M in the purified particles (Figure S2D), indicating a high degree of maturation in the JE-VLPs. The concentration of the purified VLPs was estimated by enzyme linked immunosorbent assay (ELISA- see Materials and Methods). Negatively stained electron microscopy (EM) show that the JE-VLPs have rough surfaces and diameters ranging from 30 to 40 nm (Figure S2F). Purified yellow fever virus (YFV) particles had a similar appearance but were 50 nm in diameter (Figure S2G). Dynamic light scattering (DLS) analysis indicated an average diameter of approximately 40 nm for the JE-VLPs (Figure S2E), consistent with the EM data.

### JE-VLPs from insect or mammalian cells enter the endocytic pathway

**Figure 1:**
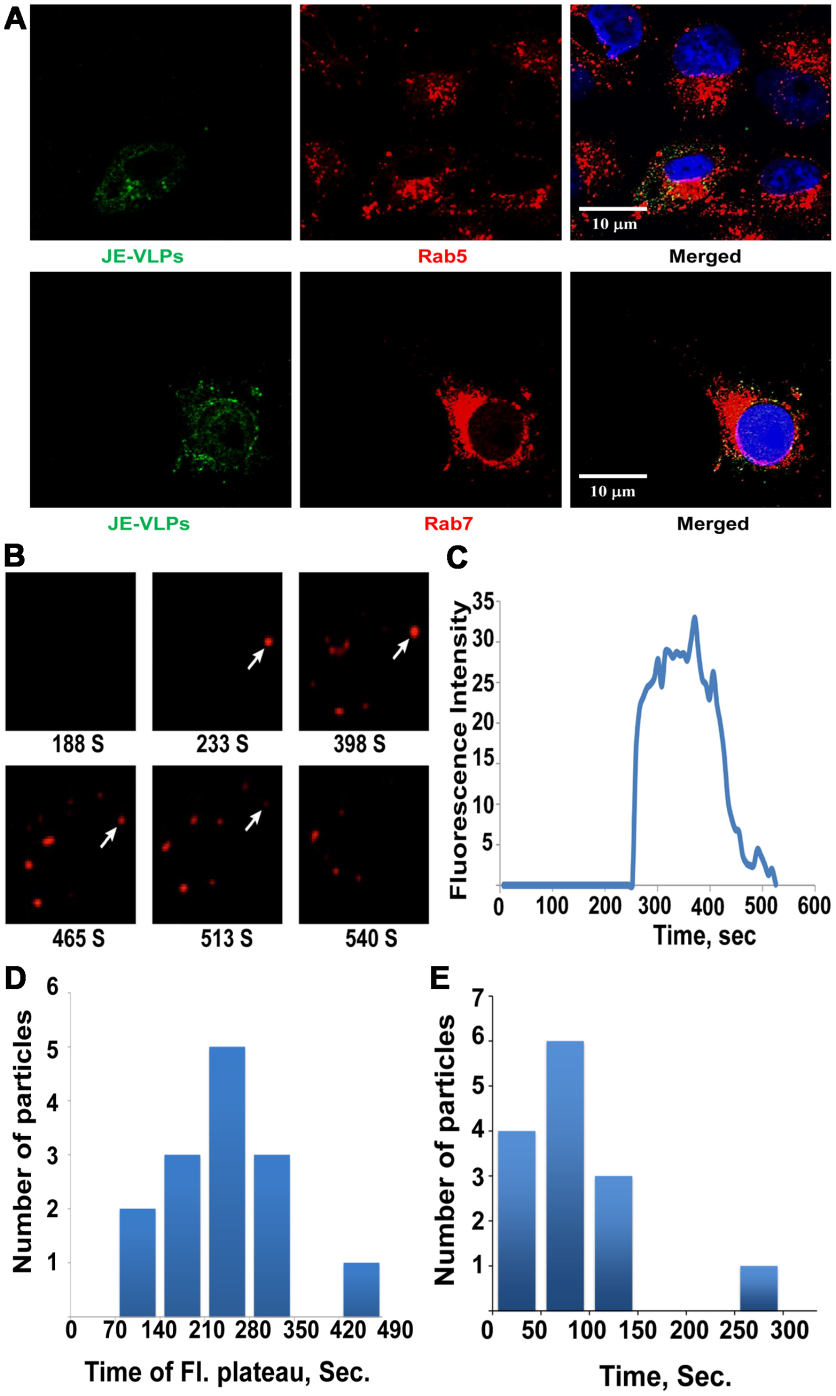
Endocytosis, live-cell tracking and membrane fusion kinetics of JE-VLPs. (A) 15 min after treatment of Vero cells with 200 µl JE-VLPs (at 17 pM or 50 ng/ml E protein), JEV E protein (green, detected with anti-West Nile primary and fluorescein-labeled secondary antibodies) colocalizes with endocytic markers Rab5 and Rab7 (red). The Pearson colocalization coefficient was 0.19 and 0.34 for E-Rab5 and E-Rab7, respectively. (B) Snapshots of JE-VLPs labeled with self-quenching concentrations of rhodamine C18 (R18) infecting live Vero cells. VLPs were tracked for 20 min after infection. Individual particles were identified and tracked with ImageJ. (C) Fluorescence intensity of the tracked particle marked with an arrow in B. Early in the endocytic pathway, approximately 5 min after infection, fusion of the viral and cellular membranes causes dilution and dequenching of the red R18 dye. R18 fluorescence remains unexpectedly stable for approximately 4 min before rapid decay due to diffusion of the dye into endosomal membranes, suggesting that the dye is transiently trapped in a small endocytic compartment. (D) Duration of R18 fluorescence, from half-maximal dequenching to half-maximal decay, for 14 tracked JE-VLPs. On average the dye remained trapped in a small endocytic compartment (with fluorescence remaining constant) for 251±97 s prior to dilution of the dye and fluorescence decay. (E) Half-time of R18 fluorescence decay (time from maximal to half-maximal fluorescence) for 14 tracked particles. The average decay halftime was 94±64 s. See also Figures S1 and S2 and Movie S1.

Upon attachment to the plasma membrane, flaviviruses localize to clathrin-coated pits and undergo endocytosis [35], [36]. Although, VLPs, like full virions, are expected to enter the endocytic pathway, this has not yet been demonstrated experimentally. We treated Vero cells, a commonly used cell line to study flaviviruses, with JE-VLPs and stained fixed cell at different time points with anti-E protein and the endosomal markers Rab5 and Rab7. VLPs attached to the Vero cells immediately. After 5 minutes, E protein colocalized with Rab5. At 15 minutes, E protein colocalized with both Rab5 and Rab7 (Figure 1A) but more so with Rab7, as indicated by the Pearson's coefficients of 0.19 and 0.34 for colocalization with Rab5 and Rab7, respectively. By 25 minutes, most of the particles colocalized with Rab7 indicating their arrival to late endolysosomal compartments. This confirms that the secreted particles follow the same general cell entry pathway as full mature virions.

### Tracking the membrane fusion of single JE-VLP and YFV particles in Vero cells

To dissect the mechanism and kinetics of JE-VLP cell entry, we tracked the membrane fusion step in real time in live cells using confocal microscopy. The lipid envelope of JE-VLPs was labeled with self-quenching concentrations of the hydrophobic dye rhodamine C18 (R18). Fusion of the VLP membrane with an endosomal membrane was detected as a sudden dequenching of R18 fluorescence as the concentrated dye was diluted with lipids from the host membrane. Fluorescent puncta that showed no dequenching were excluded from our analyses. Fusion events were first detected approximately 250 s after treatment of Vero cells, consistent with fusion occurring in early to intermediate endosomal compartments (Figure 1B–C). Notably, the R18 fluorescence signal for individual fusion events remained at its maximal level for 251±97 seconds (*n* = 14) before starting to decay and the R18 dye remained concentrated in puncta during this fluorescence plateau (Figure 1B–D). Fluorescence was expected to decay immediately after dequenching due to continued dilution with host lipids. The consistent persistence of fluorescent puncta for several minutes after fusion suggests that the R18 dye becomes trapped in an endosomal subcompartment after the initial membrane fusion event. The rate of fluorescence decay of the puncta after the fluorescence plateau, with a decay half-time of 94±64 (*n* = 14), is too great to be attributed to photobleaching alone, suggesting that the decay is due to a second and distinct event leading to dilution of the R18 dye to below detection levels (Figure 1E). Taken together the particle tracking data suggest that JE-VLPs are fusogenic, that fusion occurs in early to intermediate endosomes, and that the viral lipids remain trapped in a small endosomal subcompartment for several minutes, until a distinct event releases the lipids into a much larger compartment. R18-labeled YFV strain 17D particles produced in BHK cells using an NS1 trans-complementation strategy described previously [37] had similar R18 dequenching kinetics, as judged from analysis of a smaller number of YFV particles.

### Chloroquine inhibits flavivirus membrane fusion and delivery of viral RNA into the cytoplasm

**Figure 2:**
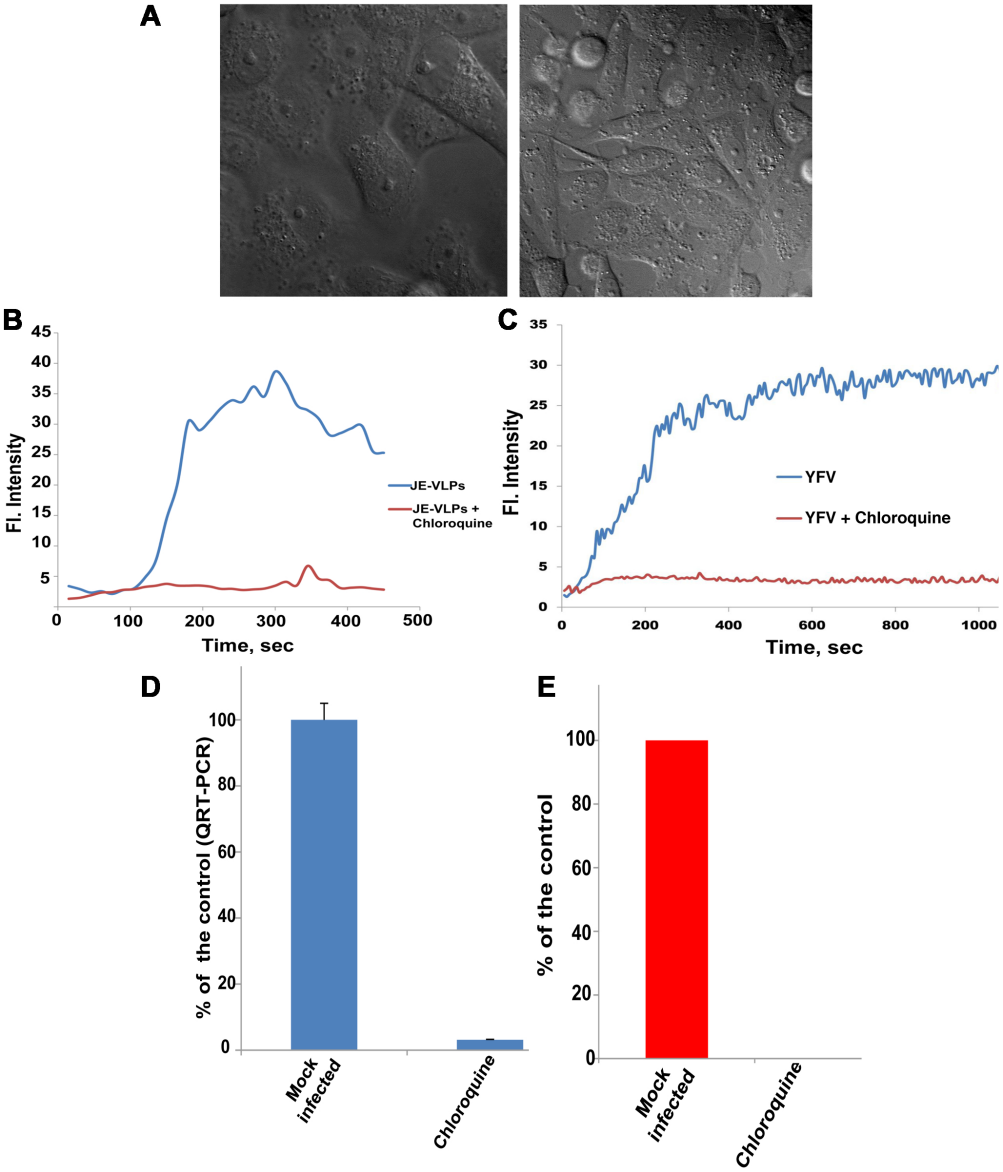
Effects on chloroquine on membrane fusion of JE-VLPs and YFV in Vero cells. (A) 0.1 g/l chloroquine (194 µM) had no notable toxic effect on the cells as indicated by differential interference contrast (DIC) microscopy of untreated cells (left) and chloroquine-treated cells (right). Cells were treated with 0.1 g/l chloroquine and infected with R18-labeled JE-VLPs (200 µl at 17 pM), (B), or with YFV (MOI = 1), (C). Chloroquine blocked VLP membrane fusion, as judged by the lack of R18 fluorescence dequenching in a field of treated cells (red curve) relative to the normal dequenching of R18 in untreated cells (blue curve). (D) Relative qRT-PCR of viral RNA in untreated (mock) and chloroquine-treated Vero cells 1 h post-infection with YFV (MOI = 1). Endosomal and cytosolic fractions were separated and total RNA was extracted from the cytosolic fraction as described in the Materials and Methods. RT-PCR was used to quantify the 3′-UTR of YFV genomic RNA. Error bars represent the standard error of the mean (SEM) of three experiments. Treatment with chloroquine reduced RNA release into the cytoplasm by >95%. (E) Plaque assay with BHK cells infect with YFV (MOI = 0.1) in presence and in absence of chloroquine. BHK cells were infected with MOI 0.1 of YFV in presence and in absence of 0.1 g/l chloroquine. Chloroquine treatment completely inhibited YFV replication. See also Figure S3.

Chloroquine is a widely used lysosomotropic drug that acts by inhibiting the acidification of the endocytic pathway. Clinical studies have demonstrated the safety, tolerability, and efficacy of chloroquine as a treatment against enveloped RNA viruses [38]. Treatment with 194 µM (0.1 g/l) chloroquine was not toxic to Vero cells (Figure 2). In the presence of chloroquine, fluorescence dequenching of R18 in R18-labeled JE-VLPs and YFV (Figure 2B–C) was completely inhibited, indicating that membrane fusion of JE-VLPs is dependent on the acidic pH of endosomal compartments. This is consistent with the inhibitory effect of chloroquine and other endosomal pH-neutralizing agents reported in other flaviviruses [35], [39].

To confirm that mature flavivirus virions are also dependent on acidic endosomal pH, and that chloroquine inhibits not only fusion but also nucleocapsid delivery into the cytoplasm, we measured the effect of chloroquine on viral RNA release and infectivity of YFV. To measure delivery of YFV genomic RNA into the cytoplasm, infected Vero cells were fractionated into cytoplasmic and endosomal fractions (Figure S3), and viral RNA in the cytoplasmic fraction was detected by relative quantitative RT-PCR (Figure 2D). Chloroquine blocked 95–97% of YFV RNA release in Vero cells. Moreover, in a plaque assay for YFV infectivity in BHK cells, chloroquine completely inhibited infectivity (Figure 2E). These results confirm that the acidity of endosomal compartments is required for flavivirus infection, and that infection can be blocked with lysosomotropic drugs such as chloroquine that raise the endosomal pH.

### Microtubules are required for RNA delivery into the cytoplasm and viral infectivity but not membrane fusion

**Figure 3:**
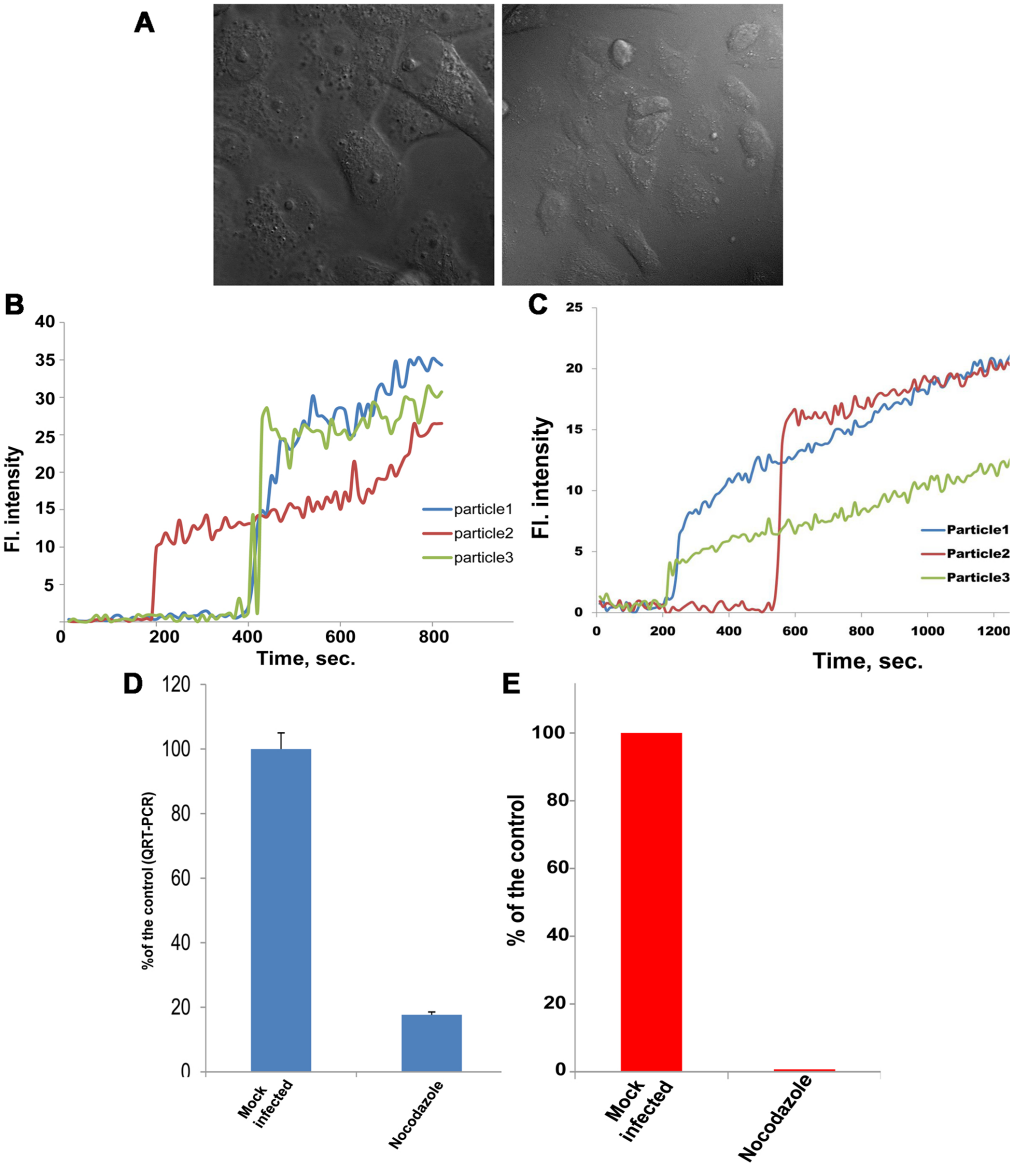
Microtubules are required for RNA delivery into the cytoplasm and viral infectivity but not membrane fusion. Cells were pretreated with 20 µM nocodazole (a microtubule polymerization inhibitor) for 1 h before treatment with R18-labeled JE-VLPs or YFV. (A) DIC micrograph of the treated cells showing that nocodazole has no toxic effect on Vero cells although the cell morphology is altered. (B) JE-VLPs (200 µl at 17 pM) and (C) YFV (MOI = 0.1–1) fused normally in Vero cells in the presence of 20 µM nocodazole, as indicated by the R18 fluorescence dequenching profiles of three representative tracked particles. (D) qRT-PCR nucleocapsid delivery assay showing that nocodazole reduced YFV nucleocapsid delivery into the cytoplasm of Vero cells by approximately 80% relative to untreated cells. Error bars represent the standard error of the mean (SEM) of three experiments. (E) Plaque assay showing that nocodazole treatment inhibited YFV replication, reducing the number of viral plaques by >97%. See also Movie S2.

In mammalian cells, endosomal carrier vesicles (ECVs) are transported from early endosomes (EEs) to late endosomes on microtubules. ECVs then dock onto and fuse with late endosomal membrane [40]. Inhibition of microtubule-dependent transport with the microtubule depolymerizing agent nocodazole inhibits West Nile virus infection [35]. Treatment with 20 µM nocodazole was not toxic to the cells, although changes in cell morphology were observed in the treated cells (Figure 3A). In cell pretreated with nocodazole, fluorescence dequenching of R18 in R18-labeled JE-VLPs and YFV occurred with similar kinetics as in untreated cells (Figure 3), indicating that nocodazole does not affect membrane fusion. However, the R18 fluorescence intensity gradually increased after fusion (Figure 3B–C), rather than decaying after a 3–4 min plateau as in untreated cells (Figure 1C–D). The gradual increase in fluorescence in the presence of nocodazole may be attributed to sequential homotypic fusion events with other non-fluorescent ECVs in early endosomal compartments. The resulting vesicles would be still relatively small (hence the lack of dilution-dependent decay) but would allow additional R18 dequenching (hence the gradual increase in fluorescence). Additionally, inefficient lipid mixing in the presence of nocodazole may contribute to the gradual increase in fluorescence. Consistent with this interpretation, YFV RNA release into the cytoplasm, measured by RT-PCR as described above, was inhibited by 80% in the presence of nocodazole (Figure 3D). Moreover, virus infectivity was completely inhibited by nocodazole (Figure 3E). Together, these results indicate that certain flaviviruses fuse with ECVs, and that membrane fusion and RNA delivery into the cytoplasm are two distinct events.

### A lipid specific to late endosomes is required for RNA delivery into the cytoplasm and viral infectivity but not membrane fusion

**Figure 4:**
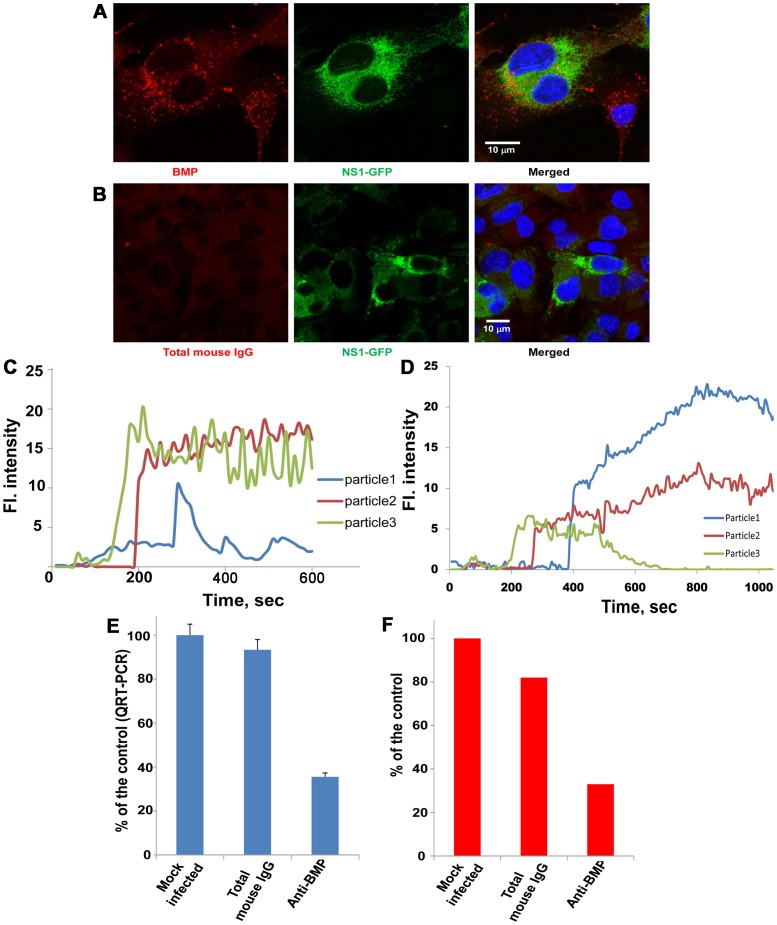
BMP, a lipid specific to late endosomes, is required for nucleocapsid delivery into the cytoplasm and viral infectivity but not membrane fusion. Cells were cultured overnight in media containing 50 µg/ml anti-BMP antibody. Cells were washed and the medium was changed before immunostaining, treatment with R18-labeled JE-VLPs (200 µl at 17 pM) or with YFV (MOI = 0.1–1). (A) Immunostaining of BMP in BHK cells. Left: BMP staining (Texas Red) was mainly perinuclear. Center: NS1-GFP (green) expression in BHK cells to transcomplement the ΔNS1-YFV genome with NS1 for viral production as described in Ref. [37]. (B) BHK cells treated with total mouse IgG instead of anti-BMP antibody. No antibody staining is detectable in the cells (left panel). JE-VLPs, (C), or YFV, (D), fused normally in Vero cells pretreated with anti-BMP antibody, as indicated by the R18 fluorescence dequenching profiles of three representative tracked particles. Error bars represent the standard error of the mean (SEM) of three experiments. (E) qRT-PCR assay showing that pretreatment of Vero cells with anti-BMP antibody reduced the delivery of YFV nucleocapsid into the cytoplasm by approximately 70% relative to untreated cells or cell treated with total mouse IgG. (F) Plaque assay showing that pretreatment of BHK cells with the anti-BMP antibody partially inhibited YFV replication, reducing the number of viral plaques by approximately 65% relative to the controls.

Having established that membrane fusion occurs early in the endocytic pathway whereas the YFV nucleocapsid is delivered into a late endosomal compartment, we sought next to determine the importance of factors specific to late endosomal compartments for fusion and nucleocapsid delivery. BMP (also known as LBPA) is an anionic lipid that is present in internal membranes, but not the limiting membrane, of late endosomes. An antibody against BMP accumulates on these internal membranes [41] and interferes with the protein sorting and membrane transport functions of the late endosome. Treatment with anti-BMP antibody causes a phenotype characteristic of Niemann-Pick disease type C (NPC) [11], [42]. To assess the role of late endosomal trafficking in flavivirus cell entry, we incubated Vero cells with an anti-BMP antibody and assay membrane fusion activity, RNA delivery and infectivity. The staining patterns of endocytosed anti-BMP antibody and of a total mouse IgG control in BHK cells are shown in Figure 4. Pretreatment with anti-BMP antibody followed by infection with R18-labeled JE-VLPs or YFV produced similar R18 fluorescence profiles as in cells pretreated with nocodazole, with normal R18 dequenching kinetics but no fluorescence decay (Figure 4C–D). We conclude that membrane fusion is not inhibited by blocking the protein and lipid sorting functions of late endosomes. In contrast, the endocytosed anti-BMP antibody reduced both YFV RNA delivery to the cytoplasm of Vero cells and YFV infectivity in BHK cells by 35% (Figure 4E–F). These data suggest that BMP in internal late endosomal membranes is required for virus infectivity, either to ensure correct ECV trafficking, or possibly to promote “back-fusion” of ECVs to the limiting membrane of the late endosome.

### PI(3) kinase activity is required for RNA delivery into the cytoplasm and viral infectivity but not membrane fusion

**Figure 5:**
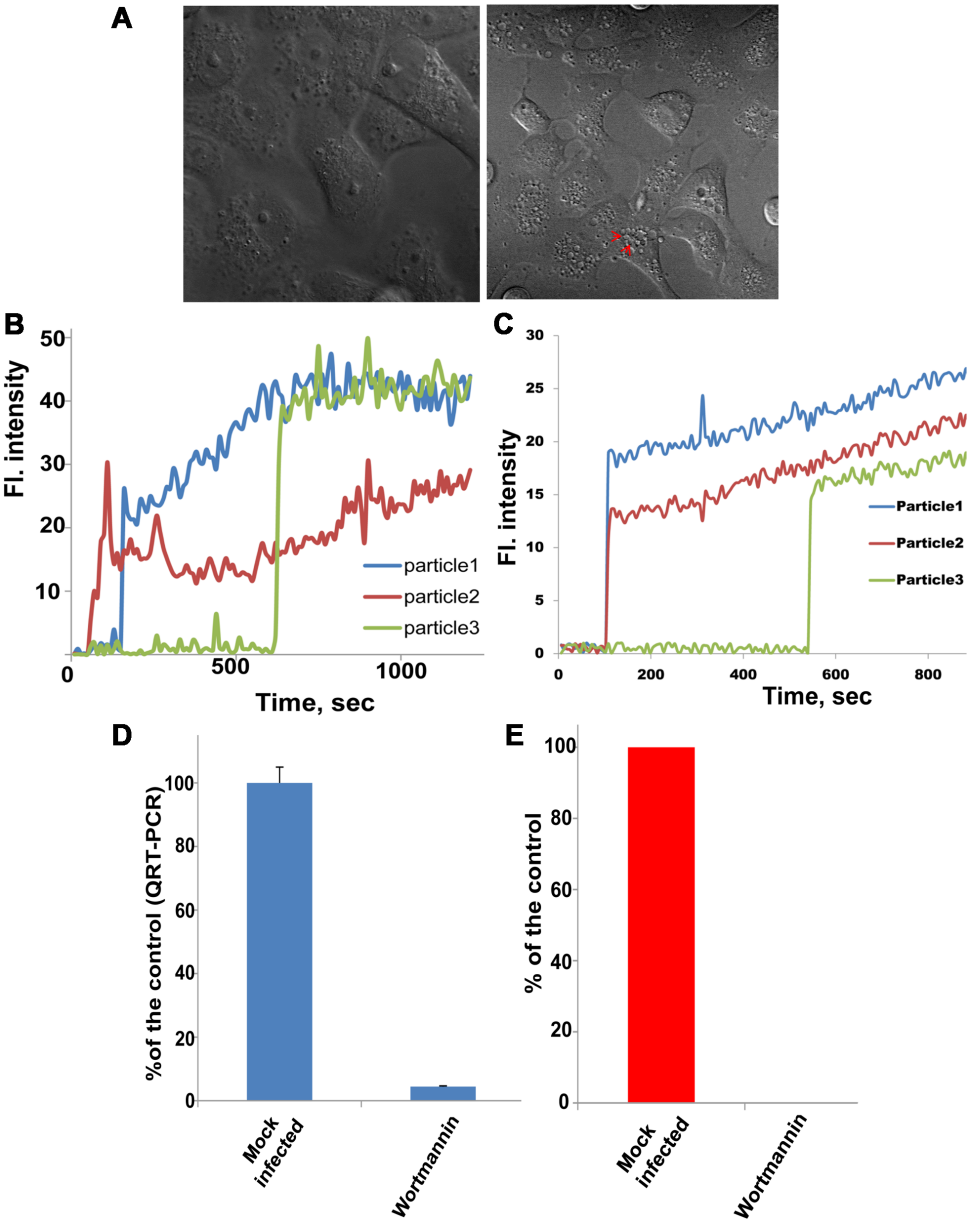
PI(3) kinase activity is required for RNA delivery into the cytoplasm and viral infectivity but not membrane fusion. Cells were pretreated with 0.1 µM wortmannin for 1 h prior to treatment with JE-VLPs (200 µl at 17 pM) or infection with YFVs (MOI = 1). (A) DIC micrograph of the treated cells showing that wortmannin had no toxic effects although treated cells showed a characteristic increase in the number of intracellular vacuoles (red arrows). JE-VLPs, (B), or YFV, (C), fused normally in Vero cells pretreated with wortmannin, as indicated by the R18 fluorescence dequenching profiles of three representative tracked particles. (D) qRT-PCR assay showing that pretreatment of Vero cells with wortmannin reduced the delivery of YFV nucleocapsid into the cytoplasm by 95% relative to untreated cells. Error bars represent the standard error of the mean (SEM) of three experiments. (E) Plaque assay showing that pretreatment of BHK cells with wortmannin reduced YFV replication and viral plaque formation to background levels. See also Movie S3.

In our emerging model of flavivirus cell-entry, virions fuse with ECVs and the nucleocapsid is delivered into the cytoplasm when the ECVs fuse back to the limiting late endosomal membrane. To test this model, we set out to evaluate the importance of factors required for ECV formation for fusion and nucleocapsid delivery and for trafficking of ECV to the late endosome. The lipid phosphatidylinositol-3-phosphate (PI(3)P) is generated by PI(3)P kinase and is required for endocytic trafficking [43]. Vero cells infected with either fusogenic or chemically inactivated YFV or JE-VLPs induced robust activation of PI(3)P kinase as indicated by phosphorylation of AKT, also known as protein kinase B (Figure S4). The PI(3)P kinase inhibitor wortmannin inhibits ECV formation in mammalian cells [44]. PI(3)P is abundant in ECVs and early endosomes, but not in the late endosome [45]. To determine whether PI(3)P kinase activity is required for membrane fusion, we pretreated Vero cells with 60 nM wortmannin and tracked fusion of R18-labeled JE-VLPs and YFV as described above. This concentration of wortmannin was not toxic but did cause vacuoles to form inside the cells as expected (Figure 5A). The resulting R18 fluorescence profiles were similar to those with nocodazole or anti-BMP antibody pretreatment, with the same R18 dequenching kinetics but no fluorescence decay (Figure 5B–C and Movie S3). Since wortmannin inhibits ECV formation, we attribute the gradual increase in fluorescence in the presence of wortmannin to inefficient lipid mixing. RT-PCR analysis and plaque assay showed that wortmannin pretreatment blocked RNA release into the cytoplasm of Vero cells and virus infectivity in BHK cells, respectively (Figure 5D–E). Collectively, these results suggest that in the absence of ECVs, JE-VLPs and YFV fuse with other as yet unidentified membranous structures or compartments, where the nucleocapsid remains trapped. Alternatively, the lipid composition or curvature of the limiting early endosomal membrane may preclude full membrane fusion of JE-VLPs. The observed R18 dequenching may then be attributed to transient hemifusion of the viral and endosomal membranes, which would allow lipid mixing of the proximal viral and endosomal lipid monolayers, and therefore dilution of R18, without nucleocapsid delivery into the cytoplasm.

### YFV and JE-VLPs bind to phosphatidylserine and fuse with liposomes with an EE/ECV-like lipid composition

**Figure 6:**
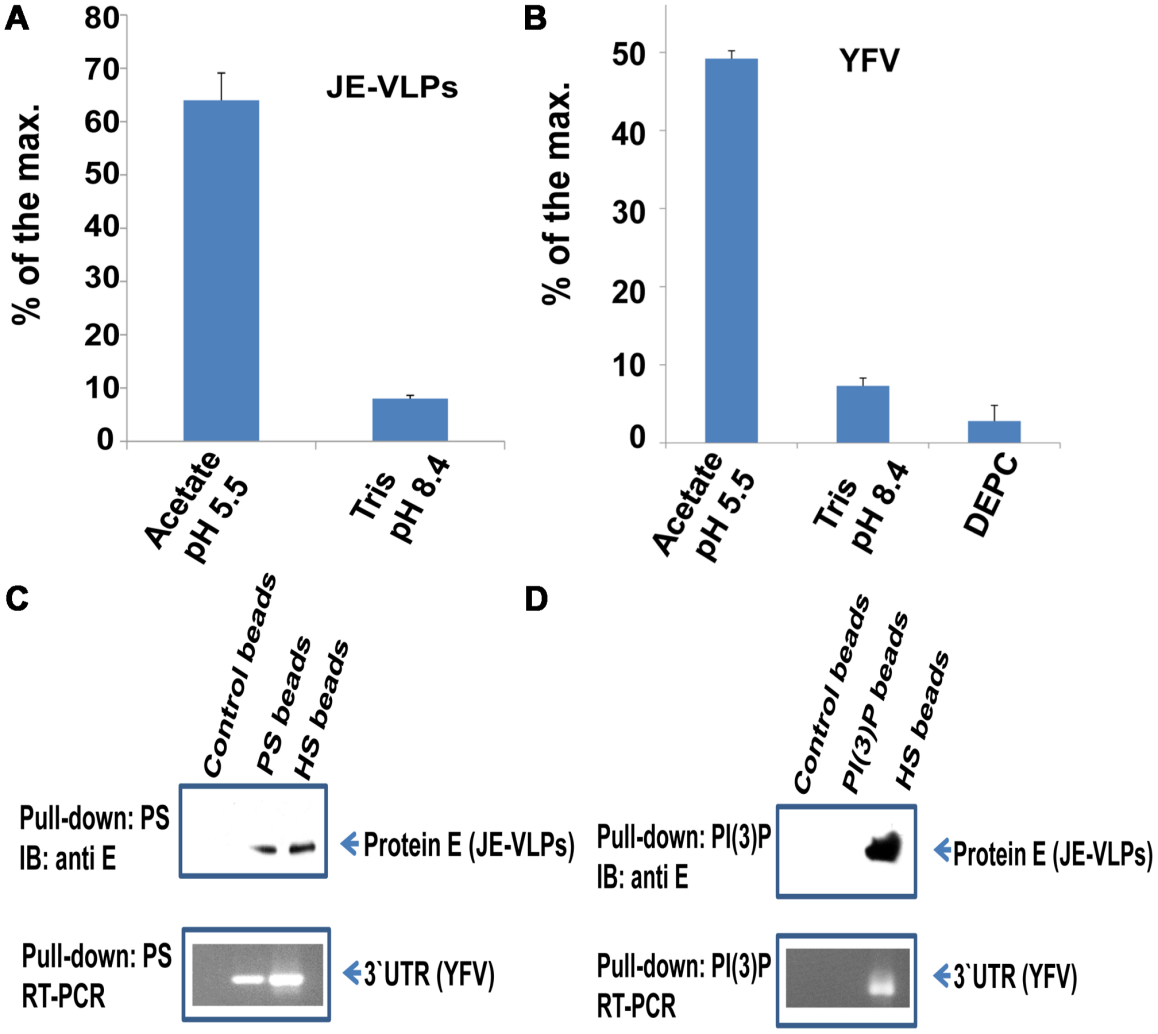
JE-VLPs and YFV bind to phosphatidylserine and fuse with liposomes with a lipid composition similar to early endosomes and ECVs. (A) Liposomes were produced from an ECV-like lipid mixture (3∶1∶1∶1∶4, Chol∶PE∶PI(3)P∶PS∶PC) as described in the Materials and Methods. JE-VLPs fused efficiently with the liposomes at pH 5.5 but not at pH 8.4. (B) YFV fused normally with the synthetic liposomes at pH 5.5 but pretreatment of the virus with 2 mM DEPC for 30 min blocked fusion at pH 5.5. Error bars represent the standard error of the mean (SEM) of three experiments with fluorescence measured in triplicate for each experiment. (C) Beads coated with the anionic lipid PS pulled down JE-VLPs and YFV. Beads coated with heparan sulfate (HS) were used as a positive control and uncoated beads were used as a negative control. (D) Beads coated with PI(3)P did not bind to either JE-VLPs or YFV. HS-beads and uncoated beads were used as positive and negative controls, respectively.

In certain flaviviruses low pH does not appear to be sufficient to trigger fusion [21]. We note that YFV fusion is only partially inactivated by a pretreatment under conditions (pH 6.2) but that infection is nevertheless completely inhibited in acidic media (Figure S5). To determine the minimal physicochemical requirements for membrane fusion of JE-VLPs and YFV, we measured fusion of R18-labeled virus particles *in vitro* in a bulk fusion assay with synthetic liposomes. The liposomes were 0.1 µm in diameter (see Materials and Methods) and their lipid composition was chosen to correspond to those in EEs/ECVs: cholesterol, phosphatidylcholine (PC), phosphatidylethanolamine (PE), PI(3)P, and phosphatidylserine (PS) at a molar ratio of 3∶4∶1∶1∶1 [7], [9]. Fusion was measured by R18 dequenching. We found that both JE-VLPs and YFV fused with the liposomes at pH 5.5, but not at neutral pH (Figure 6A). In flaviviruses, a conserved cluster of histidine side chains acts as a “pH sensor”, which triggers the fusogenic conformational change in response to the reduced pH of the endosome [16], [17]. The histidine modifying agent diethylpyrocarbonate (DEPC) inactivates VSV [46] and dengue virus [22] by inhibiting the fusogenic conformational change. We found that DEPC also blocked fusion of R18-labeled YFV with liposomes at pH 5.5 (Figure 6B). In conclusion, synthetic liposomes with a lipid composition similar to ECVs are sufficient to induce flavivirus fusion *in vitro* at low pH.

PS and PI(3)P are abundant in mammalian EEs/ECVs [7]. We found that JE-VLPs and YFV bound to PS-coated beads (Figure 6C). Similarly, heparan sulfate beads also bind the virus particles, consistent with reports that flaviviruses bind heparan sulfate through the viral envelope protein [34], [47]. However, the virus particles did not bind to PI(3)P beads (Figure 6D), suggesting that the binding to PS is not due to nonspecific electrostatic interactions and that PS may act as an intracellular receptor or fusion cofactor for flaviviruses. PS and PI(3)P beads bound with equal affinity to polyarginine peptides, indicating that the surface charges of the two types of beads are comparable (Figure S6).

Calcium released into the cytoplasm during viral infection can result in the translocation of PS from cytoplasmic to extracellular lipid leaflets [48], [49], [50]. Imaging of cells with the calcium-dependent dye Fluo-4 showed that calcium is released into the cytoplasm within one minute of infection with YFV or JE-VLPs (Figure S7). To determine whether intracellular calcium release promotes flavivirus infection we measured the effect of the cell-permeable calcium chelator BAPTA on YFV infectivity. BAPTA reduced YFV infectivity to less than 5% of the untreated infected control (Figure S7C). We propose intracellular calcium release during flavivirus infection may cause a redistribution of PS towards extracellular or luminal leaflets, which may be important for flavivirus infectivity.

### A single-chain variable region fragment of Antibody 11 (scFv11) protects Vero cells from infection by JE-VLPs but not YFV

**Figure 7:**
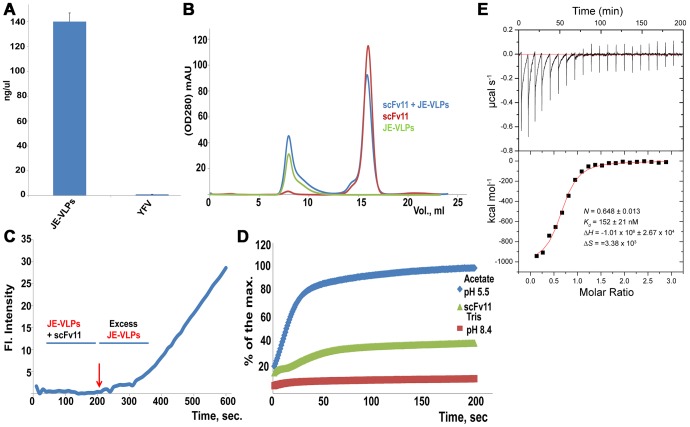
A single-chain variable region fragment of neutralizing anti-West Nile virus E antibody 11 (scFv11) binds JE-VLPs but not YFV *in vivo* and *in vitro*. (A) ELISA of JE-VLPs and YFVs treated with scFv11. The antibody fragment bound to JE-VLPs but not YFV. Error bars represent the standard error of the mean (SEM) of three experiments. (B) Size-exclusion chromatography was used to confirm the binding of scFv11 to JE-VLPs in solution. VLPs and scFv11-VLP complexes eluted in the void volume (8 ml); free or excess scFv11 eluted at 16 ml. (C) The R18 fluorescence intensity of a field of cells treated with purified scFv11-JE-VLP complexes was tracked by confocal microscopy. No dequenching was observed until free JE-VLPs were added (red arrow at 200 s). (D) Pretreatment of R18-labeled JE-VLPs with 1 µM scFv11 reduced the membrane fusion activity of the VLPs with synthetic ECV-like liposomes (see Figure 6) at pH 5.5 by approximately 50%. (E) Isothermal titration calorimetry (ITC) was used to determine the equilibrium dissociation constant of scFv11 from JE-VLPs, (*K*_d_ = 152±21 nM, and the stoichiometry of scFv11 binding to JE-VLPs, 0.648±0.013 scFv11 molecules per E protein. See also Figure S8.

Antibodies that inhibit fusion by targeting the fusion loop of the E protein are important determinants in the humoral response to flavivirus infection [51], [52], [53]. The therapeutic scFv11 antibody fragment, which recognizes the fusion loop, was selected by phage display for binding to West Nile virus E protein [54], [55]. scFv11 protects mice from a lethal challenge of West Nile virus and also protects against dengue virus types 2 and 4 [54]. To probe the reactivity of scFv11 against other flaviviruses, we used an ELISA assay to measure the binding of scFv11 antibody to either JE-VLPs or YFVs. scFv11 bound tightly to JE-VLPs but did not bind to YFV (Figure 7A). These results were confirmed by co-elution of scFv11-VLP complexes in size-exclusion chromatography (Figure 7B), and by a plaque assay with YFV showing that scFv11 had no effect on YFV infectivity.

The location of the scFv11 epitope in the fusion loop of E suggests that scFv11 inhibits viral membrane fusion [55]. To test whether scFv11 inhibits fusion of JE-VLPs, we used the *in vivo* and *in vitro* fusion assays described above. Preincubation of R18-labeled JE-VLPs with scFv11 inhibited membrane fusion in Vero cells, as judged by the lack of R18 dequenching. Subsequent addition of untreated JE-VLPs produced R18 dequenching as expected (Figure 7C). In the bulk fusion assay, scFv11 reduced the acid-induced fusion of JE-VLPs with EE/ECV-like synthetic liposomes by 50% (Figure 7D). These data suggest that the fusion loop of JE-VLPs is accessible to scFv11, which inhibits fusion of JE-VLPs in EEs/ECVs.

The dissociation equilibrium constant of scFv11 from JE-VLPs, measured by isothermal titration calorimetry, was at 150 nM (Figure 7E). scFv11 was previously shown to bind soluble form of West Nile E with a 5 nM dissociation constant [54]. The higher affinity for West Nile E could be due to the fusion loop epitope being partially occluded in JE-VLPs, or to differences in the amino acid sequence or structure of the non-cognate JE E and the cognate West Nile E. The stoichiometry of binding was 0.648±0.013 scFv11 molecules per E protein. Thus, if the JE-VLPs each contain 60 E proteins, as is the case in an electron microscopy structure of tick-borne encephalitis VLPs [56], each JE-VLP would be capable of binding approximately 40 scFv11 molecules. The observed substoichiometric binding of scFv11 suggests that one third of the E-protein epitopes in VLPs do not bind scFv11 either because they are not fully exposed or because they are clustered too closely together to allow full occupancy by scFv11 without steric clashes. The latter is more likely given the presumed *T* = 1 icosahedral symmetry of the VLPs, in which each E protein displays identical surface epitopes [56]. In contrast, in mature virions in the E proteins are distributed in three distinct chemical environments with slightly different surface epitopes. Two different neutralizing antibodies against dengue and West Nile viruses bind to only two thirds of the E proteins in their cognate virions [57], [58], providing precedents for the stoichiometry reported here for scFv11 binding to JE-VLPs.

## Discussion

Many enveloped viruses enter the endocytic pathway and rely on specific features of the endosomal environment, in particular the reduced pH and the lipid composition, to trigger membrane fusion and productive delivery of the viral genome into the cytoplasm. Although it has been established that flaviviruses generally undergo clathrin-mediated endocytosis [35], [36], [59], the sequence and kinetics of the steps required for cell entry remain unclear. It has been reported that approximately 20% of dengue virions fuse early in the endocytic pathway while the rest fuse in late endosomes [60], but it is unclear whether all of these fusion events lead to productive infection. We note that diphtheria toxin, a bacterial bipartite toxin complex composed of carrier and toxin subunits, inserts its carrier subunit into early and late endosomal membranes but the active toxin subunit is only delivered to the cytoplasm from early endosomes, and most of diphtheria toxin complexes are degraded in the lysosome [4].

**Figure 8:**
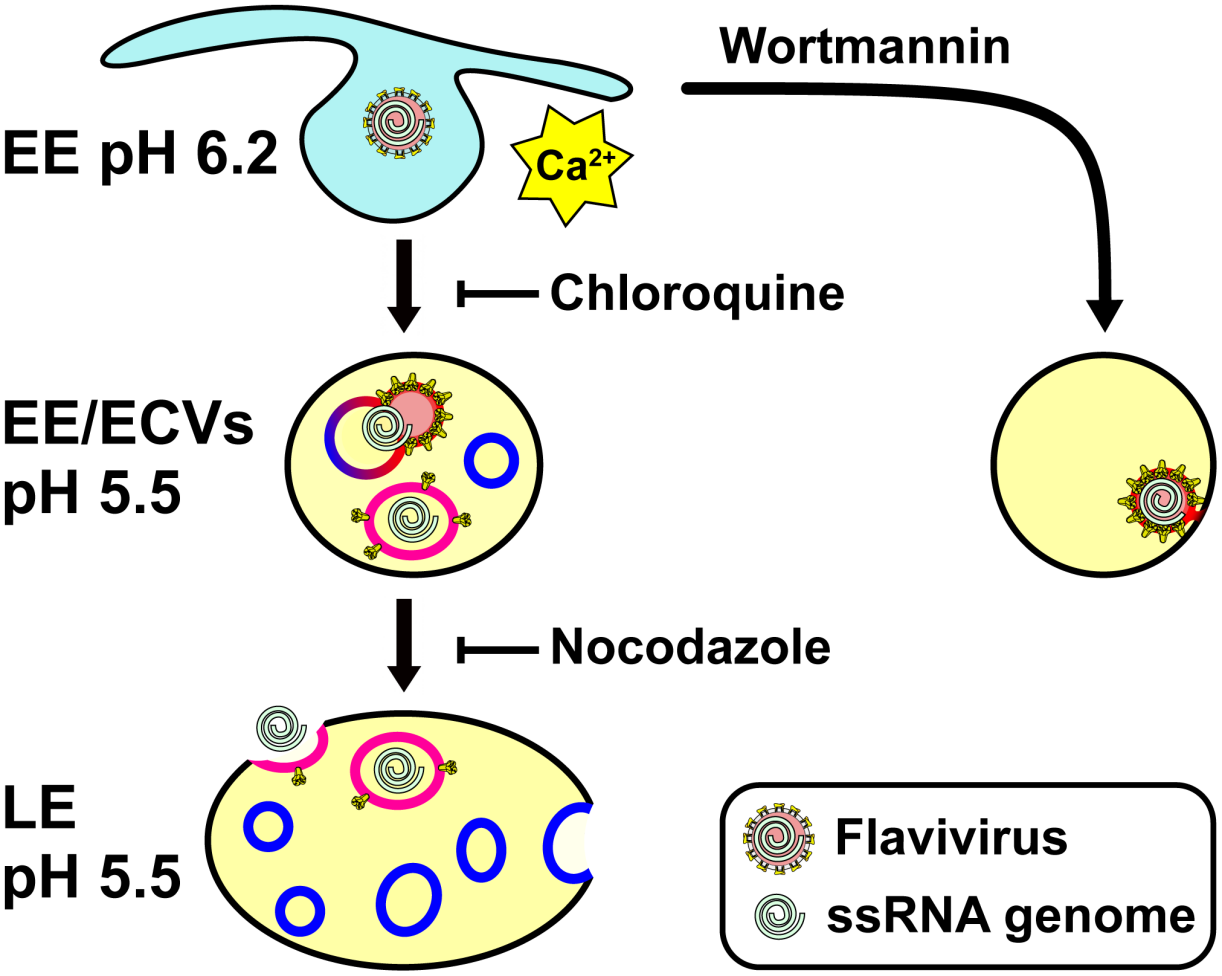
Proposed model of flavivirus cell entry. After binding to their receptors, flaviviruses enter the clathrin-mediated endocytic pathway and are directed to early endosomes (EEs). Virus entry causes an increase in cytoplasmic calcium levels, which may cause a redistribution of phospholipids in the cellular membranes. When the pH of the early endosomal compartments is reduced to approximately 6.5, about 5 min after cellular attachment, virus particles fuse preferentially with ECVs, due to the large excess of ECV membranes over the limiting endosomal membrane. The enrichment of specific anionic lipids in ECVs such as PS may further promote fusion with ECVs over fusion with the limiting endosomal membrane. The nucleocapsid remains trapped in the ECV lumen for several minutes, until the ECV fuses back with the limiting membrane of the late endosome (LE). This back-fusion event requires microtubule transport, PI(3)P-dependent signaling, the late endosomal anionic lipid BMP, and cellular fusion proteins. The PI(3)P kinase inhibitor wortmannin inhibits the formation of ECVs. In the absence of ECVs, flaviviruses either fail to fuse completely with the EE membrane (e.g. fusion proceeds only as far as hemifusion of the proximal lipid layers) or they fuse with as yet unidentified endosomal compartments in which the nucleocapsid remains trapped.

Little is known about the physical and biological properties of flavivirus VLPs- how and when they assemble, and what their possible roles are in infection and in virus evolution. In this study, we have dissected the cell entry steps of VLPs and virions from two different flavivirus species. Tracking of single virus particles in live cells and virus infectivity measurements in the presence of various cell biological inhibitors are consistent with an entry mechanism in which virus particles fuse preferentially with small endosomal carrier vesicles (ECVs), with nucleocapsid delivery into the cytoplasm occurring several minutes later, when the ECVs fuse with the limiting membrane of the late endosome. Alternatively, instead of fusing completely with ECVs, the virus particles may form metastable hemifusion intermediates with the ECVs, with full fusion only occurring in late endosomes, consistent with a previous report that dengue forms ‘restricted hemifusion’ intermediates [22]. Either way, we conclude that flavivirus membrane fusion and nucleocapsid delivery into the cytoplasm are distinct events in space and time (Figure 8).

While novel for flaviviruses, a sequential cell entry mechanism involving delivery into ECVs followed by back-fusion of the ECVs to the limiting late endosomal membrane is not unprecedented. VSV utilizes this mechanism of nucleocapsid delivery into the cytoplasm to reach the cytoplasm [5] although VSV does not impact PI(3)P signaling or trigger intracellular calcium release [31], [32], [33]. Similarly, anthrax lethal toxin (LT) from *Bacillus anthracis*, a bipartite toxin complex composed of carrier and toxin subunits, inserts carrier protein (protective antigen) into ECVs in response to endosomal acidification, and delivers its toxin subunit (lethal factor) into the cytoplasm upon back-fusion of the ECV with the limiting late endosomal membrane to deliver lethal factor to the cytoplasm [6].

The back-fusion of ECVs with the endosome's limiting membrane depends on the anionic lipid BMP [5], which is found in internal membranes and vesicles within late endosomes but not in the limiting endosomal membrane [8]. Treatment of cells with anti-BMP antibody did not inhibit membrane fusion of JE-VLPs or YFV but strongly inhibited both YFV infectivity and RNA delivery into the cytoplasm. This suggests that the cell entry mechanism of these viruses is dependent on back-fusion of ECVs to the limiting late endosomal membrane. Since anionic lipids are required for efficient fusion of dengue virus [22], the presence of the anionic lipid phosphatidylserine (PS) in ECVs may be responsible for the preference of JE-VLPs and YFV to fuse with ECV membrane over limiting endosomal membranes. Additionally, the presence of cholesterol in the target membrane promotes fusion [61], [62] and cholesterol chelation reduces flavivirus infectivity, although addition of exogenous cholesterol at the cellular attachment step has been reported to block JE and dengue virus cell entry [63]. Cholesterol is abundant in early endosomes and ECVs [8].

While anionic lipids are important in a general sense for cell entry of enveloped RNA viruses, PS may perform a more specific function. PS is abundant in early endosomes and ECVs, where PS represents about 9% of all phospholipids [7], [9]. YFV and JE-VLPs fused with liposomes of this lipid composition. Moreover, PS-coated beads bounds JE-VLPs and YFV whereas beads coated with PI(3)P, which is also anionic and present in ECVs, did not bind the virus particles. Interestingly, PS is expressed on the plasma membranes of insect cells [64] and malignant and non-apoptotic cells [65], [66].

The therapeutic antibody fragment scFv11 binds JE-VLPs with reasonably high affinity. Noninfectious flavivirus VLPs produced during infection [26] may thus serve as antibody decoys to promote immune evasion of the infectious virions. VLPs have recently been in focus as vaccine candidates [27]. Our study establishes that flavivirus VLPs can be used as a model for virus entry and for screening of therapeutic antibodies.

In summary, our work suggests a novel mechanism for flavivirus cell entry in which the virus fuses to ECVs and depends on a second cell-mediated membrane fusion event to deliver the viral genome from the vesicle lumen to the cytoplasm. We propose that flavivirus infection modulates PI(3)P-dependent signaling in the host and modifies host phospholipid distribution to promote fusion with endocytic compartments. Our findings provide a framework for future studies to determine the physicochemical basis of the preference for membrane fusion with ECVs, the nature of the contribution of specific lipids (BMP, PS, cholesterol) to fusion activity, and the precise sequence and kinetics of the molecular steps required for membrane fusion and nucleocapsid delivery.

## Materials and Methods

### Antibodies

Horse anti-WNV E antibody was a generous gift from L^2^ Diagnostics (New Haven). The scFv11 construct, a kind gift from Erol Fikrig, was expressed and purified as described [54]. The purified protein showed the expected purity and molecular weight in SDS-PAGE and size-exclusion chromatography (Figure S8). The following reagents were purchased from commercial sources: rabbit anti-human Rab5, Rab7, Phospho-AKT and Total-AKT antibodies (Cell Signaling), anti-LPBA (BMP) antibody (Echelon biosciences), Fluorescein (FITC)-labeled anti-horse antibody (Bethyl Labs.), horseradish peroxidase (HRP)-conjugated anti-horse (Sigma), Texas Red anti-rabbit antibody (Invitrogen), total mouse IgG (Sigma), Texas Red anti-mouse antibody (Invitrogen).

### Cell biological inhibitors

All inhibitors were freshly prepared before use according to the manufacturers' recommendations. 25 µM BAPTA (Sigma) was added to the virus, maintained during cellular attachment, and removed before addition of agarose plugs in plaque assays. Wortmannin (Sigma) was used at a final concentration of 0.1 µM while nocodazole (Sigma) was used at 20 µM final concentration. Chloroquine (Sigma) was used at a final concentration of 194 µM (0.1 g/l). All inhibitors were added to the cells before starting the experiments. In the plaque assay, all of the inhibitors were diluted to their half concentrations and kept in the medium after adding the agarose plugs as described in plaque assay method. Diethylpyrocarbonate (DEPC) (Sigma) was added at final concentration of 2 mM to purified YFVs for 30 min and then removed by buffer exchange using an ultrafiltration unit.

### Expression of the JEV-VLPs

The prM-E sequence of JEV strain CH2195LA was cloned into the pAcgp67 vector (BD Biosciences). Sf9 cells were co-transfected with the linearized baculovirus genome and the pAcgp67-prM-E construct. The secreted recombinant baculovirus encoding the prM-E sequences was amplified in Sf9 cells. For protein expression, Tni cells (Expression Systems) were infected with the baculovirus in ESF921 medium (Expression Systems) at 27°C. Alternatively, JE-VLPs were expressed in HEK293T cells transiently transfected with the pcDNA-prM-E mammalian expression vector. Cell media was clarified by centrifugation at 4°C. We then added 2.5% (w/v) NaCl slowly with continuous stirring, followed by 10% (w/v) polyethylene glycol 6000 (PEG6000) to precipitate the VLPs. VLPs were pelleted at 20,000 rpm for 20 min. The pellet was resuspended in 20 ml of buffer (50 mM Tris pH 8.4, 0.1 mM EDTA, 0.15 M NaCl) and centrifuged at 20,000 rpm for 2 min to remove excess PEG6000. The VLPs were then purified on a 10–40% sucrose gradient by centrifugation for 9 h at 30,000 rpm in a SW32 rotor at 4°C. The gradient was then separated into 1-ml fractions and VLPs were detected by immunoblotting. Horse anti-WNV E antibody and anti-horse secondary antibody conjugated to HRP were both used at a dilution of 1∶10^4^. Fractions containing VLPs were concentrated in ultrafiltration units with a 30-kDa molecular weight cutoff (Millipore). Buffer was exchanged for all positive layers to 50 mM Tris pH 7.4, 0.14 M NaCl and the VLPs were checked for the purity by SDS-PAGE on 4–20% acrylamide gels with Coomassie staining. The hydrodynamic radius of the JE-VLPs was determined by dynamic light scattering analysis (Malvern).

### Production of YFV in baby hamster kidney (BHK) cells

Generation of the BHK cell line expressing the NS1-GFP fusion protein for trans-complementation of YFV 17D genome lacking NS1 has been described previously [37]. Non-infectious ΔNS1 viruses were collected from the cell culture medium. The YFV particles were purified by PEG6000 precipitation and sucrose gradient as described above for JE-VLPs. In the absence of an effective antibody to detect YFV structural proteins, YFV was quantified in fractions from the sucrose gradient as plaque-forming units in a plaque assay with the BHK-NS1-GFP cells (see below).

### Plaque assay of YFV

Serial dilutions of YFV were added to BHK cells (5×10^5^ cells/ml) in DMEM medium. Viruses were allowed to attach to the cells for 1 h at 37°C. A 1∶1 mixture of 2× DMEM medium (Gibco) and autoclaved 1.6% (w/v) agarose (37°C) was then layered onto the cells in 6-well plates. After 2–3 days, the wells were fixed with 7% formalin (Sigma) and the agarose plugs were removed. Cells were stained with 0.5% crystal violet (Sigma) to visualize the plaques. Excess stain was removed with water. For acid pretreatment of YFV, the buffer was exchanged to 50 mM HEPES pH 6.2 with an ultrafiltration device (Millipore), viruses were allowed to attach to cells for 15 min in DMEM pH 6.5 or 7.4, and cells were then washed with DMEM pH 7.4 before proceeding to the next step of plaque assay.

### Negative staining

5 µl of JE-VLP suspension was applied for 2 min on the carbon surface of a glow-discharged carbon-coated grid (Microscopic Science). Excess sample was removed using absorbent paper and the grid was air-dried before examination. Data were collected using a Zeiss EM900 electron microscope.

### R18 labeling of JE-VLPs

JE-VLPs were labeled with Rhodamine C18 (R18; Invitrogen). The dye was added to the PEG-precipitated fraction of either YFV or JE-VLPs at a final concentration of 20 ng/µl and incubated for 15 min before the sucrose gradient centrifugation step.

### Time-lapse confocal microscopy

Vero cells were grown on a coverslip petri dish (MatTek) overnight at a density of 5×10^5^ cells/ml at 37°C, 5% carbon dioxide. Before microscopic examination, the medium was changed to serum-free OptiMEM (Gibco) and cells were stained with Hoechst stain (Invitrogen). JE-VLPs were added from a stock at 17 pM (50 ng/ml E protein) and the particles were kept in the medium during data collection. Time-lapse confocal microscopy was performed using a Zeiss microscope connected to 37°C incubated and buffered with 5% CO_2_. Time-lapse images were collected using a slice of 4 µm to avoid changes in confocal planes during data collection. Images were collected every 10 s. Data were analyzed with ImageJ [67].

### Immunostaining

Immunostaining was performed as described [68]. Briefly, Vero cells were grown on a coverslip overnight at 5×10^5^ cells/ml and treated at different time points with 34 pM JE-VLPs (50 ng/ml E protein). 1 µl of Hoechst stain (10 g/l) was added to the cells for 10 min at 37°C. Cells were fixed with 4% paraformaldehyde and permeabilized with 1% Triton ×100. Cells were then blocked for 1 h with 10% fetal bovine serum (FBS) and stained with the primary anti-Rab5/7 antibody according to the manufacturer's recommendation (Cell Signaling). The cells were washed 10 times with PBS. For BMP staining, cells were fed 50 µg/ml anti-BMP antibody overnight and the cells were washed and fixed as above. The cells were then permeabilized with 0.3% Tween-20 in PBS and blocked for 1 h in 10% FBS. Cells were blocked for 1 h with 10% FBS and then stained with secondary antibody conjugated to Texas Red (Invitrogen). Cells were washed 10 times with PBS and mounted with fluoromount G (Microscopic Science) before examination. Semi-quantitative colocalization analysis (Pearson coefficient calculation) was performed with ImageJ.

### Bulk fusion assay

Cholesterol, phosphatidylethanolamine (PE), PI(3)P, phosphatidylserine (PS), and phosphatidylcholine (PC) were mixed in chloroform at a molar ratio of 3∶1∶1∶1∶4, respectively, and then dried with argon gas and under vacuum for 2 h. The lipids were resuspended in 3 ml TEA buffer (10 mM triethanolamine pH 8.3, 0. 14 M NaCl) and subjected to 10 freeze-thaw cycles using liquid nitrogen and a 37°C water bath. The lipid suspension was then extruded through 0.1 µm membranes 21 times with a lipid extruder (Avanti Polar Lipids). The liposome suspension was added to R18-labeled virus particles. After a 5 min incubation, the pH was modified with either sodium acetate pH 5.5 buffer or with Tris pH 8.4 buffer (70 mM final concentration). R18 fluorescence was monitored after 1 min. with a QuantaMaster cuvette-based spectrofluorometer (Photon Technology International) or a time-domain plate-based fluorimeter (HoriBa).

### ELISA

ELISA plates were coated with 0.1 M carbonate buffer pH 9.6 and either JE-VLPs or YFV overnight at 4°C. 6×10^10^ JE-VLPs (300 ng E protein) or 6×10^7^ plaque forming units of YFV were added to each well. The coated wells were blocked with 10% FBS in PBS for 1 h at room temperature. Primary antibody was added and plates were incubated for 1 h at room temperature followed by 10 washes with PBS. Secondary antibody was added and the plate was incubated for 30 min at room temperature. Plates were then washed 10 times with PBS. HRP substrate TMB (Sigma) was added and stop solution (Sigma) was used to stop the color development. Absorbance was measured at 450 nm. Standard curves of JE-VLPs were generated by serial dilution of purified VLPs. The concentration of E protein was estimated by comparing Coomassie staining on SDS-PAGE with staining from known concentrations of bovine serum albumin (Sigma). The E protein concentration was used to determine the concentration of JE-VLPs in this study, assuming 60 copies of E per VLP.

### Pull-down assays and genomic RNA extraction

1 ml of media from BHK cells infected with a YFV multiplicity of infection (MOI) of 0.1, or insect cells expressing prM-E were mixed with a 50 µl suspension of beads coated with heparan sulfate (HS; Sigma), phosphatidylserine (PS; Echelon biosciences), or phosophoinisitol-3-phosphate (PI(3)P). The beads were pre-equilibrated with 50 mM Tris pH 7.4 and 0.1 M NaCl. Uncoated beads (Echelon biosciences) were used as a negative control. The beads were collected by centrifugation, washed with equilibration buffer and eluted with 50 mM Tris pH 7.4 and 0.5 M NaCl. Samples containing JE-VLPs were concentrated and the buffer was exchanged to 50 mM Tris pH 7.4, 0.14 M NaCl using an ultrafiltration unit (MilliPore). Samples were then analyzed by SDS-PAGE on 4–20% acrylamide gels and analyzed by Western blotting using the anti-WNV-E antibody. For samples containing YFV, RNA was extracted from the bead eluates with Trizol (Invitrogen) in the presence of 50 µg/ml yeast transfer RNA (RNase and DNase free; Sigma) as a viral RNA carrier. RNA was quantified by RT-PCR as described below. To control for the surface charges of the PI and PI(3)P beads, we tested binding of 2.5 µg/µl polyarginine (5–15 kDa, Sigma) to each types of bead, using 100 µl beads. The binding, wash, and elution steps for polyarginine were performed as described above for JE-VLPs and YFV. Polyarginine in the eluate was quantified using Bradford reagent.

### Subcellular fractionation of cytosolic and endosomal fractions

We employed an established protocol to isolate endosomes from the cytosolic fraction [5], [11], [41], [69]. Briefly, Vero cells (6×10^6^ cells/ml) in DMEM were infected with YFV (MOI = 1) and incubated for 1 h. Cells were grown in 2 g/l HRP (Sigma) for 40 min post-infection. Cells were washed with PBS and harvested by centrifugation at 1500 rpm for 5 min at 4°C. Cells were suspended in homogenization buffer (3 mM imidazole and 8.5% sucrose pH 7.4 plus protease inhibitors (Roche)) and passed through a steel syringe needle 20 times. The nuclear fraction was isolated by centrifugation for 10 min at 1 kg and 4°C. Sucrose was added to the post-nuclear supernatant (PNS) to a final concentration of 40% (w/v). This mixture was overlaid with 35%, 27%, and 8.5% sucrose cushions in 10 mM HEPES pH 7.4. Samples were centrifuged for 1 h at 100 kg in a SW60 Ti swinging-bucket rotor. Late endosomes were collected at the 27/8.5% sucrose interface while early endosomes were collected at the 35/40% interface. Total RNA was extracted with Trizol (as described above) from the load (cytosolic) fraction. The purity and integrity of the purified RNA was determined by OD260/280 and by 1% formaldehyde agarose gel electrophoresis. To confirm that the isolated endosomes were intact, infected cells were grown with HRP for the last 20 min of the infection and the HRP activity of the endosomal or cytosolic fractions was measured after lysis with 1% Tween-20 (Figure S3A). Effective separation of the cytosolic and endosomal fractions was also confirmed by Western blotting with antibodies against Rab5 and Rab7 (Figure S3C).

### RT-PCR and qRT-PCR

The 3′ untranslated region downstream primer (3UTR, bases 10109–10128: 5′-AACCCACACATGCAGGACAA-3′) and glyceraldhyde-3-phosphatedehydrogenase downstream primer (GAPDH, bases 1157–1175: 5′-TCCACCACCCTGTTGCTGT3′) were used to reverse-transcribe the viral RNA and cellular housekeeping gene GAPDH, respectively. Quantitative real-time PCR (qRT-PCR) was performed using the same downstream primers and the 3UTR upstream primer (10337–10318 bases, 5′-GTTGCAGGTCAGCATCCACA-3′) and GAPDH upstream primer (bases 724–742: 5′-ACCACAGTCCATGCCATAC-3′). PCR reactions were carried out in triplicate using an RT-PCR kit (Roche) and an ABI 9700HT RT-PCR instrument (Applied Biosystems). The amplified products (228 bp for 3UTR and 450 bp for GAPDH) were identified on 2% agarose gels. RT-PCR products were relatively quantitated with the software SDS. Endogenous GAPDH was used as a control for the quality of the total extracted RNAs. Neither 3UTR nor GAPDH formed primer dimers as judged by the dissociation curve.

### Isothermal titration calorimetry (ITC)

Binding of scFv11 to JE-VLPs was analyzed in 50 mM Tris pH 7.4, 0.14 M NaCl, 2 mM β-mercaptoethanol at 25°C, with an iTC200 calorimeter (MicroCal). The sample cell contained 3.1 µM JE-VLPs or buffer only, and the titrant syringe contained 40 µM scFv11. An initial injection of 1.5 µl of scFv11 was followed by 20 serial injections of 2.0 µl scFv11, each at 10 min intervals. The stirring speed was 1,000 rpm and the reference power was maintained at 11 µcal/s. The net heat absorption or release associated with each injection was calculated by subtracting the heat associated with the injection of scFv11 to buffer. Thermodynamic parameters were extracted from a curve fit to the data to a single-site model with Origin 7.0 (MicroCal). Experiments were performed in triplicate.

### Size-exclusion chromatography binding assay

JE-VLPs, scFv11 and VLP-scFv11 complexes were separated on a Superdex 200 10/300 GL column (GE Healthcare) in 50 mM Tris pH 7.4, 0.14 M NaCl.

### Visualization of intracellular calcium release

Vero cells were loaded with 5 µM of Fluo-4 (Invitrogen) for 15 min in DMEM medium. Cells were either infected with YFV (MOI = 5) or treated with 200 µl JE-VLPs (at 17 pM or 50 ng/ml E protein). As a positive control we used 10 mM ionomycin (Invitrogen). As a negative control we pretreated Vero cells with 25 mM BAPTA for 30 min before loading the cells with Fluo-4. Images were collected at one frame every 2 s. Fluo-4 fluorescence was analyzed with ImageJ.

### Supporting Information

Supporting Information includes seven figures and three movies and can be found with this article at the Journal's website.

## Supporting Information

Figure S1 **Expression of JE-VLPs in insect and mammalian cells.** (A) HEK293T cells were transfected with a construct for the expression of JE prM-E. Cells were grown on a coverslip for immunostaining. JE E expression (green) was detected only in cells successfully transfected with prM-E construct. (B) Tni insect cells infected with a recombinant baculovirus designed to express prM-E were plated on a coverslip and subjected to immunostaining using anti-West Nile E antibody. Cells expressing prM-E are stained in red with Texas Red anti-rabbit antibody. Hoechst stain was used to stain the nucleus (blue).
(TIF)

Figure S2 **Purification of JE-VLPs and YFV.** Purification of JE-VLPs, (A), and YFV, (B), by affinity chromatography using a heparan sulfate column. Virus particles were precipitated with PEG 6000, resuspended and loaded onto the column. A NaCl gradient was used to elute the particles (0–0.5 M for JE-VLPs and 0–2 M for YFV). (C) Dot blot detection of JE-VLPs in fractions from a 10–40% sucrose gradient. (D) Coomassie stain of VLPs purified by sucrose gradient centrifugation. Bands corresponding to pr, M and E proteins were detected (arrows). (E) Determination of the hydrodynamic radius of the purified JE-VLPs by dynamic light scattering. The JE-VLPs form a monodisperse population with a radius of approximately 20 nm. Negatively stained electron micrographs of the purified JE-VLPs, (F), and YFV, (G), using 3% uranyl acetate as the contrast agent (pH 4.2). JE-VLPs have a diameter of diameter of approximately 30 nm; YF viruses have a diameter of 50 nm.
(TIF)

Figure S3 **Isolation of intact early and late endosomes from Vero cells infected with YFV and cultured in the presence of horseradish peroxidase (HRP).** Vero cells infected with YFV (MOI = 1) for 1 h with addition of 2 g/l HRP for the last 15 min of the infection. Cells were homogenized, and the post-nuclear fraction (PNS) was separated by sucrose gradient centrifugation. (A) Quantification of HRP in the cytosolic and endosomal fractions. The cytosolic fraction contained less than 5% of the HRP activity of the endosomal fraction, indicating that endosomal membranes were mostly intact in the endosomal fraction. (B) RNA extraction from the cytosolic fraction of infected and uninfected Vero cells. The integrity of the purified RNA was assessed by the presence of intact 18S and 28S ribosomal RNA. (C) Western blot of sucrose gradient centrifugation fractions containing early and late endosomes (EEs and LEs), and cytosol using anti-Rab5 or anti-Rab7 antibodies for detection. As expected, only the late endosomal fraction was positive for Rab7. Both endosomal fractions but not the cytosolic fraction were positive for Rab5. (D) RT-PCR of the 3′ untranslated region of YFV RNA (left) and endogenous GAPDH (right) in the cytosolic cellular fraction in the presence of different inhibitors. GAPDH was a control for successful isolation of host transcripts and for potential effects of the inhibitors on the quality of the input RNA. The levels of YFV RNA were used to quantify delivery of the nucleocapsid into the cytoplasm.
(TIF)

Figure S4 **Flaviviruses activate the PI(3)P kinase signaling pathway in Vero cells.** Vero cells grown in serum-free DMEM for 30 min were treated with YFV (MOI = 1) or JE-VLPs (17 pM, or 50 ng/ml E protein). Lysates were analyzed at 15, 30 and 60 min. (A) Western blot analysis using anti-Phospho-AKT (upper panel) and Total-AKT (lower panel) antibodies. As a control, serum was added in presence or absence of 60 nM wortmannin (two leftmost lanes). (B) Western blot analysis of Vero cells treated with DEPC-inactivated JE-VLPs and YFV in presence and absence of wortmannin (W). As a positive control serum was added to the leftmost lane. Cells grown in serum-free DMEM were used as a negative control (second lane from the left).
(TIF)

Figure S5 **Acid pretreatment only partially inactivates YFV.** Plaque assay showing that acid pretreatment (incubation in 50 mM HEPES pH 6.2 for approximately 30 min) only inactivated 40% of YFV in BHK cells in DMEM pH 7.4. Addition of acid-treated YFV to BHK cells in DMEM pH 6.5 almost completely inhibited plaque formation, suggesting that acid-inactivation of YFV is partially reversible.
(TIF)

Figure S6 **PS and PI(3)P beads have a comparable ionic binding capacity.** To determine whether PS- and PI(3)- beads have comparable ionic binding capacity, we measured binding of both types of beads to polyarginine. The experiment was carried out as described for the JE-VLPs and YFV, except that polyarginine in the eluted samples was quantified with Bradford reagent. Two different types of beads bind with equal affinity to polyarginine, indicating that the surface charges of the beads are comparable. See also Figure 6A–B.
(TIF)

Figure S7 **Flavivirus infection triggers intracellular calcium release.** Vero cells were incubated with 5 µM Fluo-4 for 15 min and infected with YFV (MOI = 1). (A) Snapshots of Vero cells infected with YFV at different time points showing an increase in intracellular calcium (green). Images were collected at one frame every 2 s. (B) Kinetics of intracellular calcium release upon YFV infection. Relative fluorescence intensity is expressed as the fraction of the fluorescence observed in Fluo-4-labeled Vero cells treated with ionomycin (“% Max. Fl. Intensity”). (C) Cell-permeable calcium chelator BAPTA blocks YFV infection. BHK cells were used in a plaque assay in the presence and absence of 25 µM BAPTA.
(TIF)

Figure S8 **Purification of the recombinant scFv11 antibody fragment from** ***E. coli*** **.** scFv11 was expressed and purified as described in the Materials and Methods. (A) Size-exclusion chromatography of purified histidine-tagged scFv11 was used to assess size, purity and solubility of the protein. Most of the scFv11 migrates as a monomer (“b”). A small fraction of the protein migrates as a dimer (“a”). (B) SDS-PAGE with Coomassie staining of the purified scFv11 was used to further assess the purity of the protein. The dimer (a) and monomer (b) peaks both contain pure scFv11.
(TIF)

Movie S1 **Tracking membrane fusion of R18-labeled JE-VLPs in Vero cells, monitored as dequenching of R18 dye.** Frames were captured every 10 s. The movie frame rate is 7 frames per second.
(MOV)

Movie S2 **Tracking membrane fusion of R18-labeled JE-VLPs in Vero cells in the presence of nocodazole.** Frames were captured every 10 s. The movie frame rate is 7 frames per second.
(MOV)

Movie S3 **Tracking membrane fusion of R18-labeled JE-VLPs in Vero cells in the presence of wortmannin.** Frames were captured every 10 s. The movie frame rate is 7 frames per second.
(MOV)
