# Supplementary figures and images for: Viral Membrane Fusion and Nucleocapsid Delivery into the Cytoplasm are Distinct Events in Some Flaviviruses
Source: PLoS Pathog. 2013 Sep 5;9(9):e1003585. doi: 10.1371/journal.ppat.1003585 (PMC3764215; doi:10.1371/journal.ppat.1003585)

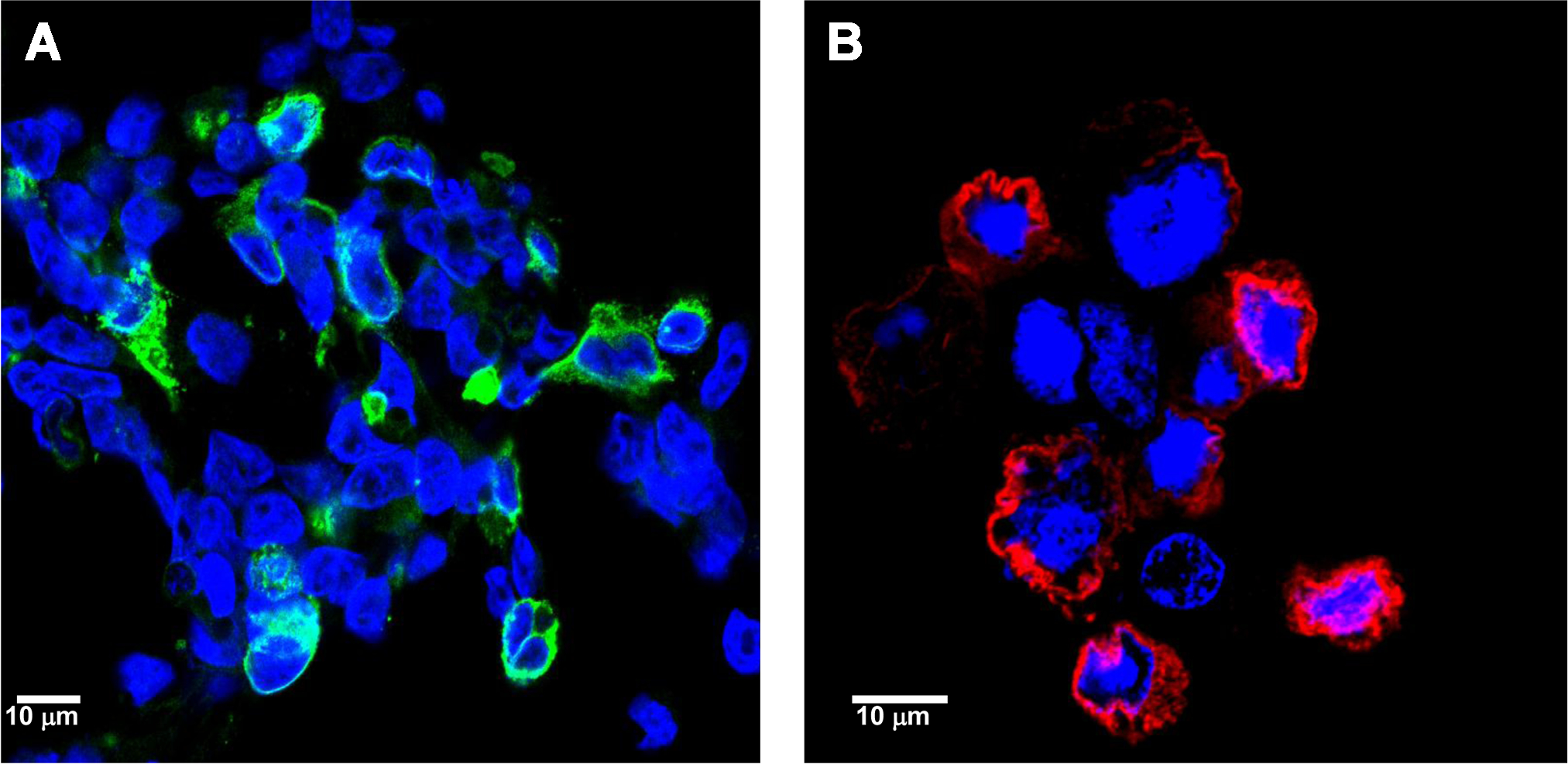

Supplement: Figure S1 — Expression of JE-VLPs in insect and mammalian cells. (A) HEK293T cells were transfected with a construct for the expression of JE prM-E. Cells were grown on a coverslip for immunostaining. JE E expression (green) was detected only in cells successfully transfected with prM-E construct. (B) Tni insect cells infected with a recombinant baculovirus designed to express prM-E were plated on a coverslip and subjected to immunostaining using anti-West Nile E antibody. Cells expressing prM-E are stained in red with Texas Red anti-rabbit antibody. Hoechst stain was used to stain the nucleus (blue). (TIF) [file ppat.1003585.s001.tif]

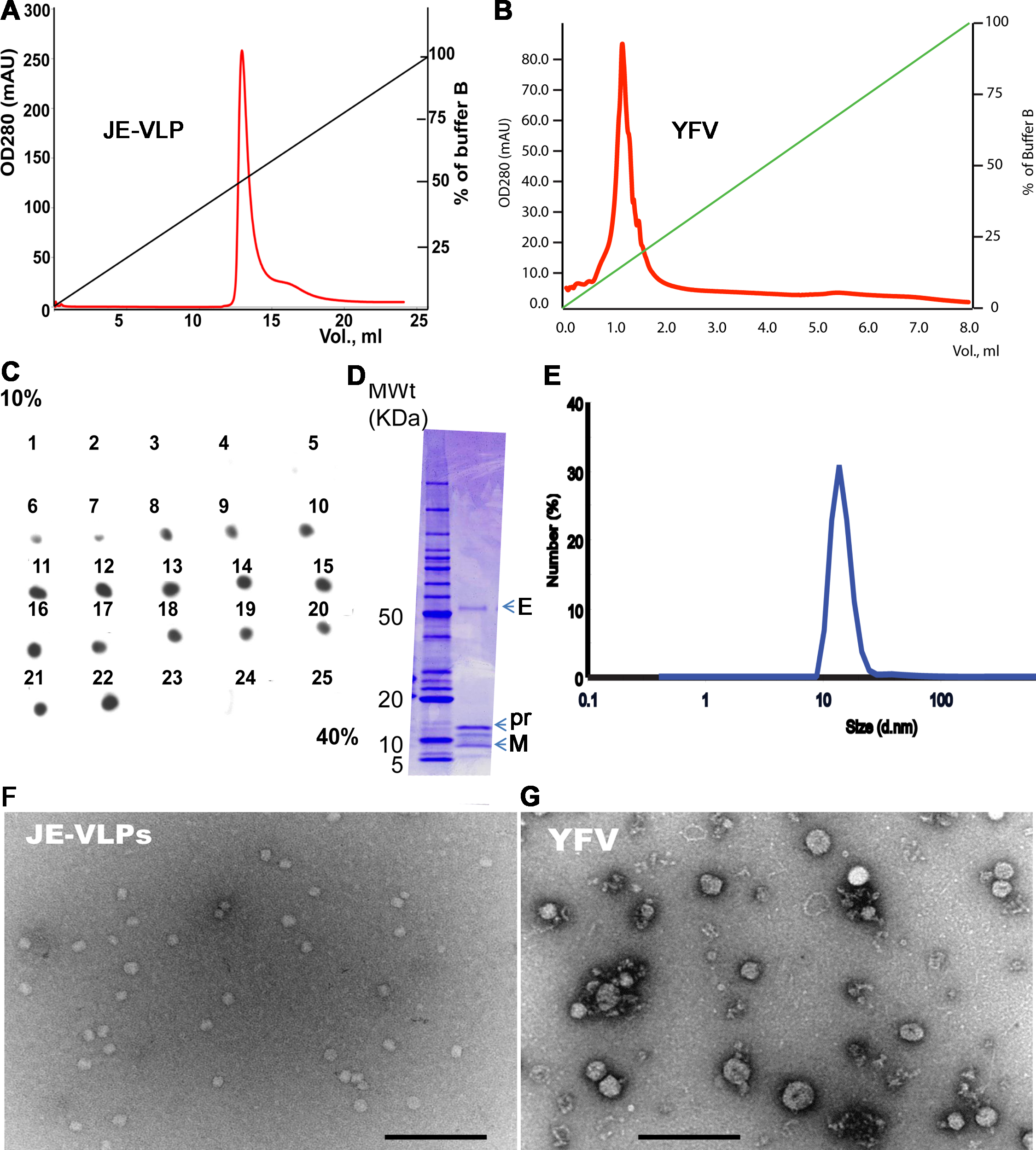

Supplement: Figure S2 — Purification of JE-VLPs and YFV. Purification of JE-VLPs, (A), and YFV, (B), by affinity chromatography using a heparan sulfate column. Virus particles were precipitated with PEG 6000, resuspended and loaded onto the column. A NaCl gradient was used to elute the particles (0–0.5 M for JE-VLPs and 0–2 M for YFV). (C) Dot blot detection of JE-VLPs in fractions from a 10–40% sucrose gradient. (D) Coomassie stain of VLPs purified by sucrose gradient centrifugation. Bands corresponding to pr, M and E proteins were detected (arrows). (E) Determination of the hydrodynamic radius of the purified JE-VLPs by dynamic light scattering. The JE-VLPs form a monodisperse population with a radius of approximately 20 nm. Negatively stained electron micrographs of the purified JE-VLPs, (F), and YFV, (G), using 3% uranyl acetate as the contrast agent (pH 4.2). JE-VLPs have a diameter of diameter of approximately 30 nm; YF viruses have a diameter of 50 nm. (TIF) [file ppat.1003585.s002.tif]

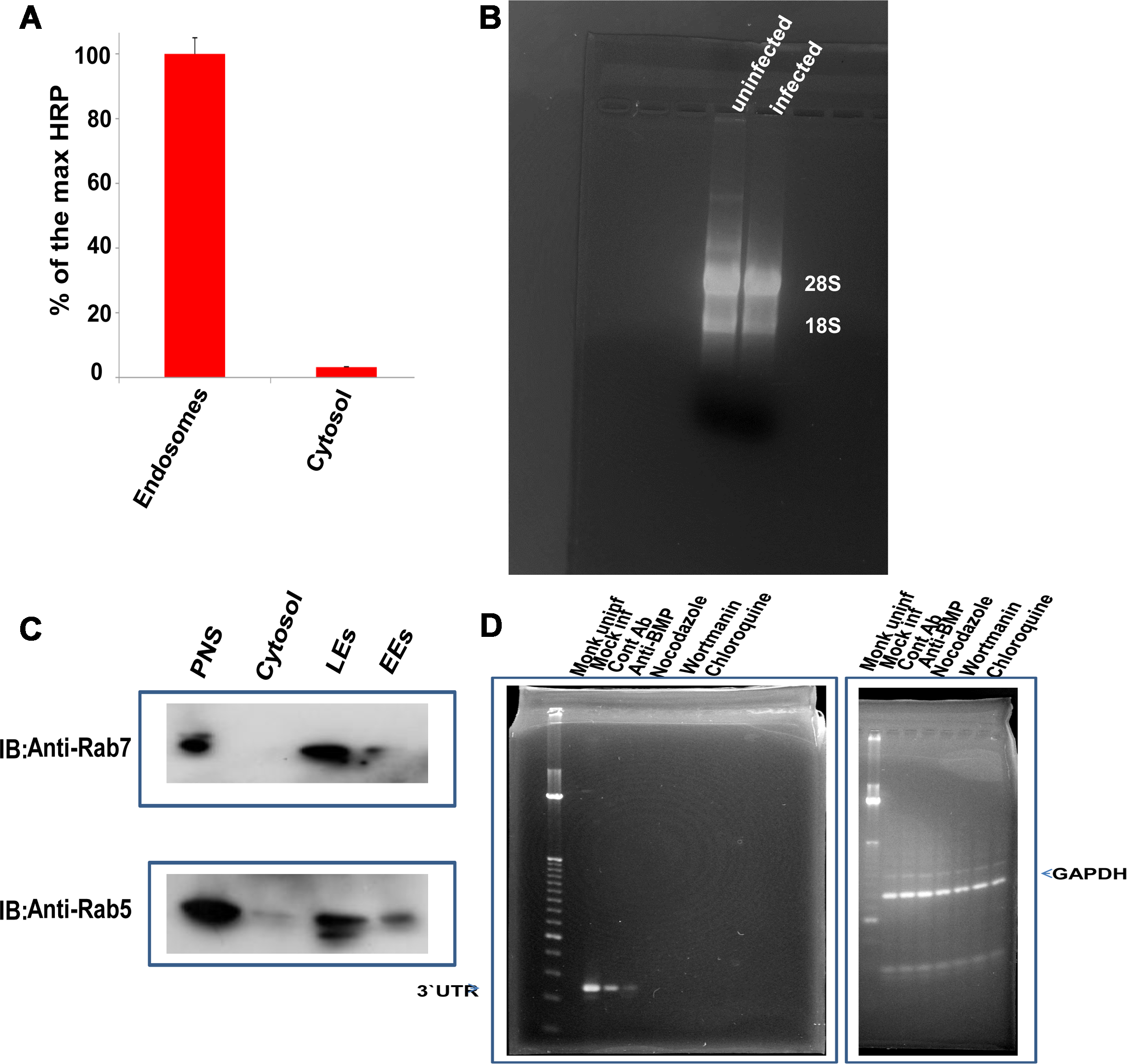

Supplement: Figure S3 — Isolation of intact early and late endosomes from Vero cells infected with YFV and cultured in the presence of horseradish peroxidase (HRP). Vero cells infected with YFV (MOI = 1) for 1 h with addition of 2 g/l HRP for the last 15 min of the infection. Cells were homogenized, and the post-nuclear fraction (PNS) was separated by sucrose gradient centrifugation. (A) Quantification of HRP in the cytosolic and endosomal fractions. The cytosolic fraction contained less than 5% of the HRP activity of the endosomal fraction, indicating that endosomal membranes were mostly intact in the endosomal fraction. (B) RNA extraction from the cytosolic fraction of infected and uninfected Vero cells. The integrity of the purified RNA was assessed by the presence of intact 18S and 28S ribosomal RNA. (C) Western blot of sucrose gradient centrifugation fractions containing early and late endosomes (EEs and LEs), and cytosol using anti-Rab5 or anti-Rab7 antibodies for detection. As expected, only the late endosomal fraction was positive for Rab7. Both endosomal fractions but not the cytosolic fraction were positive for Rab5. (D) RT-PCR of the 3′ untranslated region of YFV RNA (left) and endogenous GAPDH (right) in the cytosolic cellular fraction in the presence of different inhibitors. GAPDH was a control for successful isolation of host transcripts and for potential effects of the inhibitors on the quality of the input RNA. The levels of YFV RNA were used to quantify delivery of the nucleocapsid into the cytoplasm. (TIF) [file ppat.1003585.s003.tif]

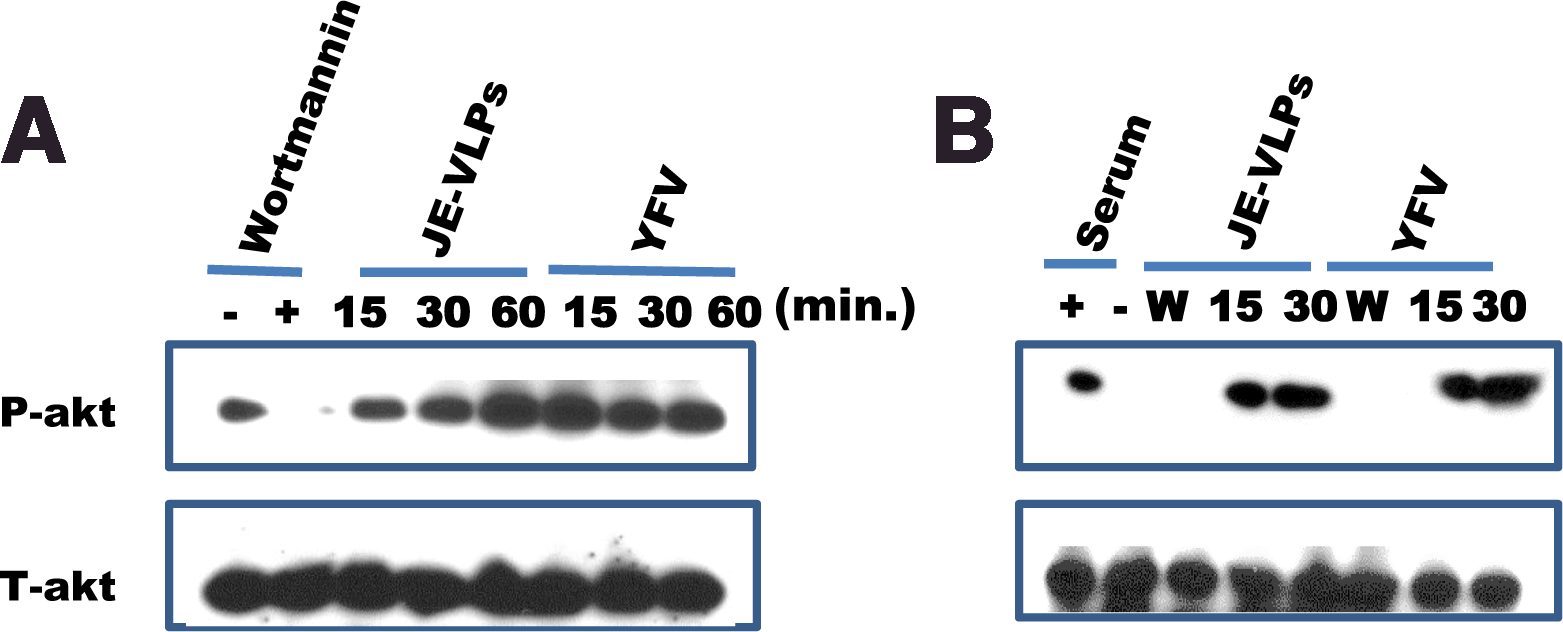

Supplement: Figure S4 — Flaviviruses activate the PI(3)P kinase signaling pathway in Vero cells. Vero cells grown in serum-free DMEM for 30 min were treated with YFV (MOI = 1) or JE-VLPs (17 pM, or 50 ng/ml E protein). Lysates were analyzed at 15, 30 and 60 min. (A) Western blot analysis using anti-Phospho-AKT (upper panel) and Total-AKT (lower panel) antibodies. As a control, serum was added in presence or absence of 60 nM wortmannin (two leftmost lanes). (B) Western blot analysis of Vero cells treated with DEPC-inactivated JE-VLPs and YFV in presence and absence of wortmannin (W). As a positive control serum was added to the leftmost lane. Cells grown in serum-free DMEM were used as a negative control (second lane from the left). (TIF) [file ppat.1003585.s004.tif]

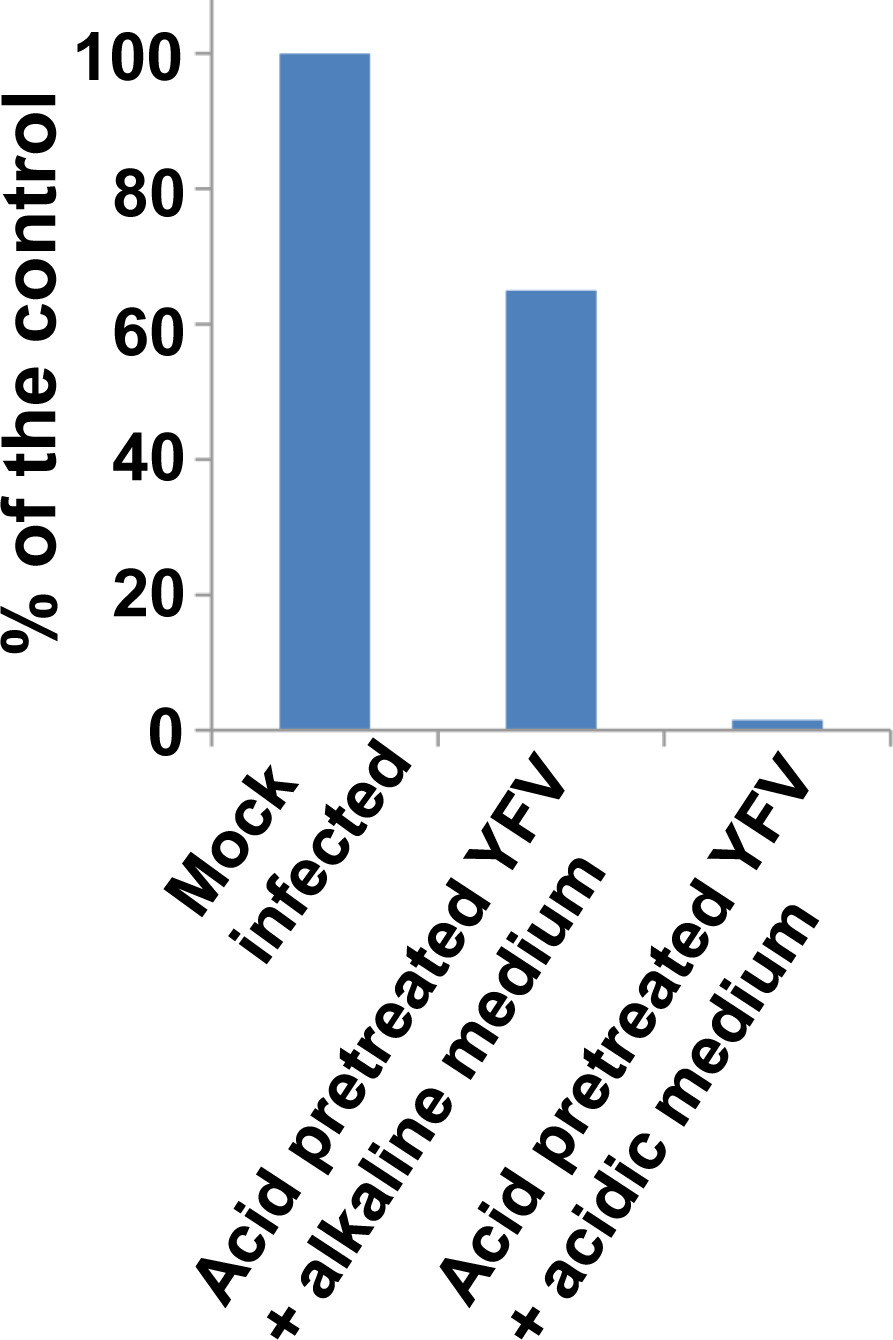

Supplement: Figure S5 — Acid pretreatment only partially inactivates YFV. Plaque assay showing that acid pretreatment (incubation in 50 mM HEPES pH 6.2 for approximately 30 min) only inactivated 40% of YFV in BHK cells in DMEM pH 7.4. Addition of acid-treated YFV to BHK cells in DMEM pH 6.5 almost completely inhibited plaque formation, suggesting that acid-inactivation of YFV is partially reversible. (TIF) [file ppat.1003585.s005.tif]

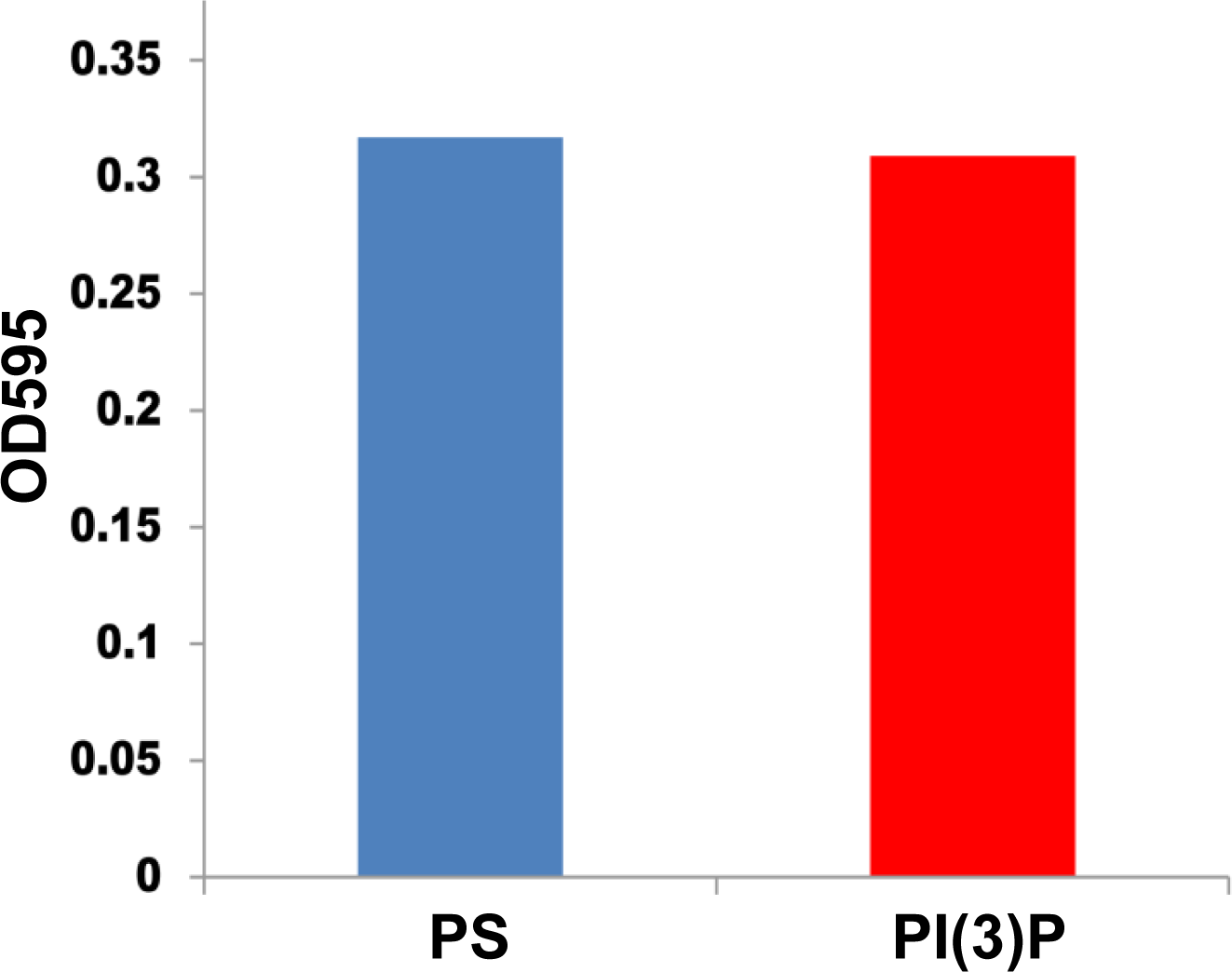

Supplement: Figure S6 — PS and PI(3)P beads have a comparable ionic binding capacity. To determine whether PS- and PI(3)- beads have comparable ionic binding capacity, we measured binding of both types of beads to polyarginine. The experiment was carried out as described for the JE-VLPs and YFV, except that polyarginine in the eluted samples was quantified with Bradford reagent. Two different types of beads bind with equal affinity to polyarginine, indicating that the surface charges of the beads are comparable. See also Figure 6A–B. (TIF) [file ppat.1003585.s006.tif]

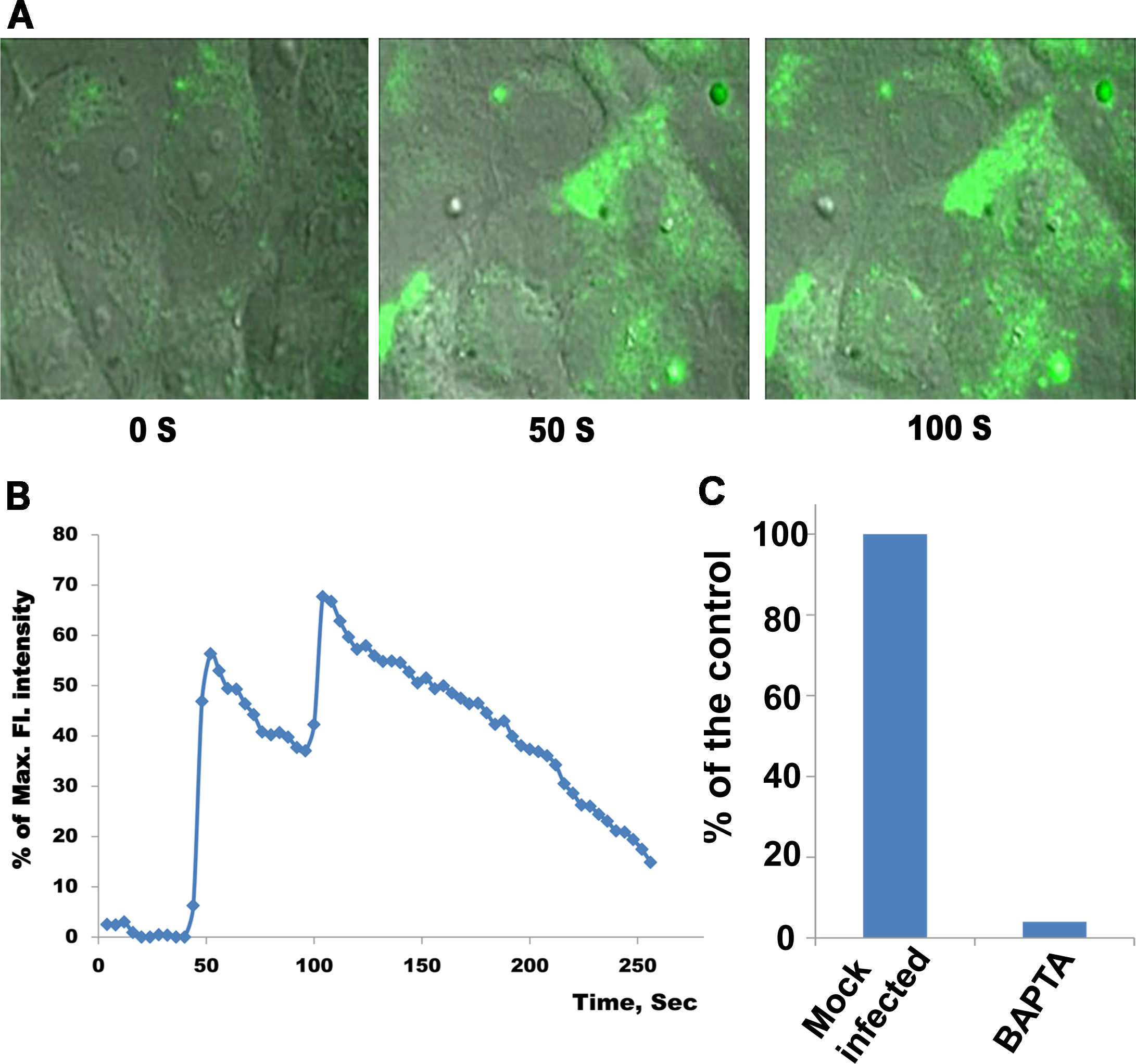

Supplement: Figure S7 — Flavivirus infection triggers intracellular calcium release. Vero cells were incubated with 5 µM Fluo-4 for 15 min and infected with YFV (MOI = 1). (A) Snapshots of Vero cells infected with YFV at different time points showing an increase in intracellular calcium (green). Images were collected at one frame every 2 s. (B) Kinetics of intracellular calcium release upon YFV infection. Relative fluorescence intensity is expressed as the fraction of the fluorescence observed in Fluo-4-labeled Vero cells treated with ionomycin (“% Max. Fl. Intensity”). (C) Cell-permeable calcium chelator BAPTA blocks YFV infection. BHK cells were used in a plaque assay in the presence and absence of 25 µM BAPTA. (TIF) [file ppat.1003585.s007.tif]

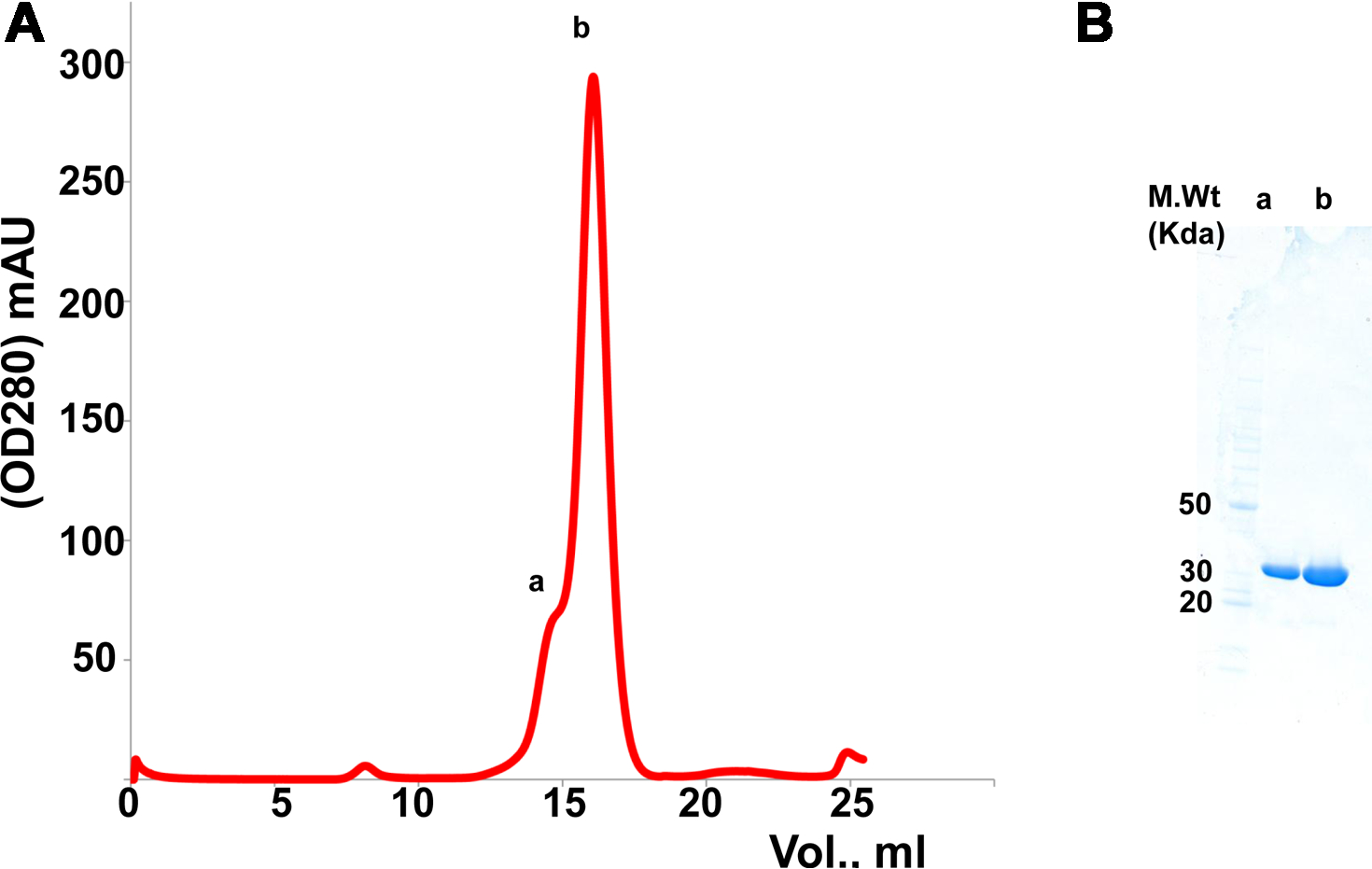

Supplement: Figure S8 — Purification of the recombinant scFv11 antibody fragment from E. coli . scFv11 was expressed and purified as described in the Materials and Methods. (A) Size-exclusion chromatography of purified histidine-tagged scFv11 was used to assess size, purity and solubility of the protein. Most of the scFv11 migrates as a monomer (“b”). A small fraction of the protein migrates as a dimer (“a”). (B) SDS-PAGE with Coomassie staining of the purified scFv11 was used to further assess the purity of the protein. The dimer (a) and monomer (b) peaks both contain pure scFv11. (TIF) [file ppat.1003585.s008.tif]
